# Synthesis of pyridone derivatives using 2D rod like bifunctional Fe based MOF and CuO nanocomposites as a novel heterogeneous catalyst

**DOI:** 10.1038/s41598-023-43045-6

**Published:** 2023-09-21

**Authors:** Negar Hoot, Enayatollah Sheikhhosseini, Sayed Ali Ahmadi, Mahdieh Ghazizadeh, Moslem Malekshahi, Mahdieh Yahyazadehfar

**Affiliations:** 1 Department of Chemistry, Kerman Branch, Islamic Azad University, Kerman, Iran; 2 Department of Physics, Kerman Branch, Islamic Azad University, Kerman, Iran

**Keywords:** Chemistry, Materials science, Nanoscience and technology

## Abstract

In this study, a new and efficient Rod-like bifunctional Fe-based MOF@CuO nanocomposites (RL BF Fe-based MOF@CuO NC) were synthesized as new and efficient heterogeneous catalyst through a simple method from easily available 1,3,5-benzenetricarbocylic acid linker, nitrate ferric as a source of iron and copper oxide (CuO) nanoparticles under microwave irradiation. The synthesized nanocatalysts were characterized with different techniques such as Brunauer–Emmett–Teller (BET), energy dispersive spectroscopy (EDS), field emission scanning electron microscopy (FE-SEM), mapping, transmission electron microscopy (TEM), X-ray diffraction (XRD), vibrating sample magnetometer (VSM), thermogravimetric analysis (TGA) and Fourier transform infrared spectroscopy (FT-IR). The RL BF Fe-based MOF@CuO NC had relatively high specific surface area (203 m^2^ g^−1^) while exhibiting superparamagnetic properties. The catalytic activity of RL BF Fe-based MOF@CuO NC was explored in a facile and green methodology to prepare diverse N‑amino-2-pyridones by one-pot four component reactions comprising aromatic aldehyde, malononitrile, methyl cyanoacetate and hydrazine hydrate within mild and solvent-free conditions. This protocol enjoys features like providing the final products during low reaction times in excellent yields under solvent-free conditions. The use of easily available and inexpensive reactants for the synthesis of the catalyst, environmental compatibility, low catalyst loading, fast and clean work-up and reusability of catalyst for several cycles with consistent activity are counted as the outstanding features of this procedure.

## Introduction

Multicomponent reactions (MCRs) are becoming important tools for the efficient and rapid synthesis of new diverse complex products in chemistry, especially in pharmaceuticals and drug discovery with attractive biological properties^1–4^. The advantages of MCRs over conventional linear syntheses that cause MCRs being systems of extremely eco-friendly and ideal including, solvents, reagents, time and energy saving, quick and simple implementation, bond-forming efficiency, no production of by-products, minimize waste, lower costs, high degree of atom economy since most of the atoms of substrates will present in the target product, avoidance of complex purification procedure, high total yield and high selectivity^1–10^. The combination the merits of MCRs with the advantages of solvent-free conditions and easily recovered heterogeneous catalyst can meet the requirements of green chemistry^4^.

Metal–organic frameworks (MOFs) refer to porous hybrid materials^11^ with adjustable pore size^12^, high surface area^12^, thermal stability and good chemical^13^. They can be modified through several post-synthesis treatments or on-site methods to improve their performance^12,14^. They have gained widespread interest in various applications such as sensors^15^, storage or gas separation^15^, removal of pesticides^16^ and organic pollutants^17^, electronic and optical devices^18^, drug delivery^15^ and biomedical purposes^19^. MOFs and their nanocomposites have advanced catalytic features^20^.

The high surface-to-volume ratio of these structures results in promoted the activity of nanoparticles, shorter reaction time, enhanced selectivity, and convenient recovery from reaction mixtures using uncomplicated filtration techniques^21,22^.

In turn, MOFs have catalytic features but the introduction of metal oxides enhances the catalytic efficiency of MOF to the next level^23^. In addition, metal oxides have very active sites and high electronic conductivity but the low surface area restricts the direct use of metal oxides as catalyst. On the other side, MOFs usually have high surface area but poor electronic conductivity and low active site. Thus, the composite materials uniting the MOFs with metal oxides can provide the increased surface area as well as active metal sites. Thus, the synergetic action of both the MOF and metal oxides overcomes the shortcoming of individual frameworks and makes the bridge between these materials^24^.

Copper oxide (CuO) is an intermediate metal oxide with a narrow band gap (1.2–1.4 eV). It is a promising p-type material whose physicochemical properties strongly depend on its size and morphology^11,12^. Various morphologies of CuO nanostructures such as nanosheets, hollow-spherical, nano-needles, nanofibers, nanowires, nanofilaments have been utilized in multidimensional applications including catalysis, sensors, magnetic storage media, Li batteries, field emission, solar energy transformation, electronics and semiconductors^25,26^.

Recently, the synthesis of one-dimensional (1D) nanostructures has attracted considerable attention because 1D nanostructures have potential applications in wide-ranging sectors including catalysis, sensing, electronics and photoelectronics, with performances that are anticipated to be superior to those of their bulk counterparts^27,28^. Besides, the different numbers of active sites and crystal plane effects explained why the nanorods showed higher activity than the corresponding nanoparticles^29,30^.

The presence of versatile synthons of N-Amino-2-pyridone in complex biological scaffolds has resulted in a vast range of biological and pharmaceutical properties.

Among a large diversity of heterocyclic compounds, the synthetic development of 2-pyridone derivatives has currently changed into an interesting challenge and an ongoing field of concern owing to the presence this valuable synthons in natural compound, which can lead to the synthesis of various nitrogen-containing heterocyclic structures, including *β*-lactam, quinolizidine, pyridine, piperidine, as well as indolizidine alkaloid^31^ with a wide spectrum of significant biological and pharmacological properties and physiological activities such as antibacterial^32^, antifungal^33^, antitumor^34,35^, cardiotonic^36^, psychotherapeutic^37^, and possible HIC-1 specific transcriptase inhibition^38^.

Ful synthetic intermediates of 1,6-diamino-2-oxo-4-phenyl-1,2-dihydro pyridine-3,5-dicarbonitrile are an N‑amino-2-pyridone subset, representing a combination of valuable synthons of 2-oxo-3-cyanopyridines and 3,5-dicyanopyridines with divers and significant biodynamic, biological and pharmaceutical properties which led to this type of pyridine derivatives with high reactivity and vast medicinal utility with a wide spectrum of synthetic N-heterocycles, including 1,2,4-triazolo[1,5-a]pyridines^39^, pyrazolo[4,3-e][1,2,4]triazolo[1′,5′-a]pyridines^40^, pyrido[1,2-b][1,2,4]triazines^39,41^, as well as pyrido[1,2-b][1,2,4]triazepines^39^.

Considering the significance of 2-pyridone skeleton, modern organic chemical sciences seek to develop effective, novel, and eco-friendly synthesis protocols for preparing 1, 6-diamino-2-oxo-4-phenyl-1,2-dihydropyridine-3,5-dicarbonitrile derivatives.

A few catalysts were previously employed to synthesize N‑amino-2-pyridones such as bismuth (III) nitrate pentahydrate as an efficiently practical Lewis acid catalytic agent^42^, piperidine^43^, magnesium oxide as an extremely powerful heterogeneous base catalytic agent^42^, polyaniline^44^, K_2_CO_3_^45^ and poly(4-vinylpyridine)^44^. The above methods took the advantage of 2-cyanoacetohydrazide as the starting material to synthesize *N*-amino-2-pyridones. The current study thus sought to synthesize derivatives of 1,6-diamino-2-oxo- 4-phenyl-1,2-dihydropyridine-3,5-dicarbonitrile by the four-component one-pot coupling of aromatic aldehydes, malononitrile, methyl cyanoacetate and hydrazine hydrate using a solvent-free and reflux conditions in the presence of RL TF Fe-based MOF@CuO NC.

## Experimental section

### Materials and apparatus

High-purity chemical substances were bought from Sigma Aldrich and used with no additional purification. Physical constants of the products were compared with the authentic samples and FT-IR spectroscopy for threir characterization. Thin layer chromatography (TLC) on Silica G60 F254 (Merck) TLC plates was used to monitor the reaction progress and determine the substrate purity. An Electrotermal 9100 device in open capillary tubes was employed to measure melting points, the values of which were left uncorrected. FTIR spectra were recorded for KBr pellets of the samples using a JASCO FT-IR-4000 spectrophotometer. NMR spectroscopy (^1^H NMR) was obtained using a Bruker Avance 400 MHz NMR spectrometer in d_6_-DMSO at ambient temperature. The crystallinity, phase structure, and crystallite size of RL TF Fe-based MOF@CuO NC were determined by a PC-APD X-ray diffractometer and Kα radiation (α_2_, λ_2_ = 1.54439 Å) and graphite mono-chromatic Cu radiation (α_1_, λ_1_ = 1.54056 Å) (Philips, the Netherlands). The X’Pert HighScore Plus software was used for data analysis. The XRD pattern was obtained in the range 2°–80° 2θ, with a step size of 0.016°. Then, SEM and EDS (KYKY & EM 3200) were applied to investigate RL TF Fe-based MOF@CuO NC. Thermal behavior analysis was carried out in N_2_ atmosphere in the temperature range of room temperature to 350 °C using a STA-1500 thermoanalyzer. A Lakeshore (model 7407) was employed to evaluate magnetization within magnetic fields under room temperature.

## Synthesis of RL BF Fe-based MOF@CuO NC nano-catalyst

### Synthesis of CuO nanoparticles

First, 0.1 M copper (II) sulfate pentahydrate (CuSO_4_·5H_2_O) was dissolved in deionized water. Then, saturated NaOH solution was added until the pH of the solution reached 8.0; where the resulting precipitates were collected. Raw materials were removed from the obtained product by three times washing with ethanol, followed by drying at 80 °C for 12 h. The resulting precipitates were calcinated under 400 °C within a 1-h period.

### Synthesis of Fe-based metal organic frameworks (Fe-based MOF)

First, 0.1 M copper (II) sulfate pentahydrate (CuSO_4_·5H_2_O) was dissolved in deionized water. Then, saturated NaOH solution was added until the pH of the solution reached 8.0; where the resulting precipitates were collected. Raw materials were removed from the obtained product by three times washing with ethanol, followed by drying at 80 °C for 12 h. The resulting precipitates were calcinated under 400 °C within a 1-h period.

### Synthesis of bi-functional Fe-based metal organic frameworks (BF Fe-based MOF)

0.092 mmol (0.026 g) of surfactant (sodium dodecyl sulfate) was dispersed in N-hexane (solution A). Fe-MOF (0.461 mmol, 0.283 g), Fe_2_O_3_ (0.154 mmol, 0.36 g), and bentonite (0.154 mmol, 0.0344 g) were dissolved in ion-free water (solution B). Then, solution B was added to A. The obtained mixture was heated to 80 °C for 2 h. The developed MOF sediments were collected after overnight refrigeration. Raw materials were removed from the obtained product by three times washing with boiling water. Finally, the MOF precipitates were calcined for one hour in an oven at 150 °C.

### Synthesis of rod-like BF Fe-based MOF@CuO nanocomposite

Deionized water was used to disperse 3 mmol of dried TF Fe-based MOF at 80 °C in a beaker. Next, 1 mmol of CuO nanoparticles were added and the mixture was stirred for 10 min to achieve a homogeneous solution. The final powder mixture was delivered to a glassy vial, which was promptly transferred into the microwave oven (300 W) and microwaved for 60 min. Acetic acid was used to wash the obtained products, to remove the raw materials. The dried powder was finally calcined at 190 °C for 70 min^46–52^.

### The general procedure to synthesize N‑amino-2-pyridones (5a–l)

A combination of hydrazine hydrate (3 mmol, 0.15 mL), methyl cyanoacetate (3 mmol, 0.27 mL), malononitrile (3 mmol, 0.198 g), aromatic aldehydes (3 mmol), and RL TF Fe-based MOF@CuO NC (10 w%) was heated under solvent-free and reflux conditions and stirred for a determined period, as shown in Table 2. TLC was employed to monitor the reaction. Acetone was used to dissolve the resulting mixture after it was cooled. Stirring the mixture for two minutes was followed by filtering the suspended solution and recovering the heterogeneous catalyst. The pure product obtained after acetone evaporation and washing solid precipitates with water. Melting points, FTIR, ^1^H NMR and ^13^C NMR spectra were considered to examine the structural characteristics of the products.

### Selected spectral data

*1,6-diamino-4-(4-methoxyphenyl)-2-oxo-1,2-dihydropyridine-3,5-dicarbonitrile (5a)*: m.p: 220–222 °C (lit.^47^ 222–224 °C); Yield: 98%; ^1^H NMR (400 MHz, DMSO-*d*_*6*_): δ 3.62 (s, 3H, OCH_3_), 3.85 (s, 3H, OCH_3_), 5.51 (s, 2H, NH_2_), 6.66–7.24 (m, 3H, CH_Ar_), 8.85 (s, 2H, NH_2_) ppm; ^13^C NMR (100 Hz, DMSO-*d*_6_): δ 55.1, 55.5, 66.1, 105.0, 118.9, 119.4, 130.7, 132.0, 159.3, 161.4, 162.2, 164.9, 174.6. ppm.

*1,6-diamino-4-(4-chlorophenyl)-2-oxo-1,2-dihydropyridine-3,5-dicarbonitrile (5b)*: m.p: 340–343 °C (lit.^47^ 341–342 °C); Yield: 87%; ^1^H NMR (400 MHz, DMSO-*d*_*6*_) δ: 5.71 (s, 2H, NH_2_), 7.60 (d, *J* = 7.6 Hz, 2H, CH_Aro_), 7.92 (d, *J* = 7.6 Hz, 2H, CH_Aro_), 8.74 (s, 2H, NH_2_) ppm; ^13^C NMR (100 Hz, DMSO-*d*_6_): δ = 68.7, 80.6, 115.2, 116.4, 128.3, 129.1, 130.0, 132.6, 136.0, 153.8, 158.2, 160.7 ppm.

*1,6-diamino-4-(4-nitrophenyl)-2-oxo-1,2-dihydropyridine-3,5-dicarbonitrile (5c)*: m.p: > 340 °C (lit.^47^ > 340 °C); Yield: 91%; ^1^H NMR (400 MHz, DMSO-*d*_*6*_): δ 5.79 (brs, 2H, NH_2_), 7.63–8.44 (m, 4H, CH_Aro_), 8.75 (brs, 2H, NH_2_) ppm; ^13^C NMR (100 Hz, DMSO-*d*_6_): δ 53.6, 106.4, 114.9, 124.4, 131.7, 137.2, 149.2, 152.8, 153.3, 161.7 ppm.

*1,6-diamino-4-(2-fluorophenyl)-2-oxo-1,2-dihydropyridine-3,5-dicarbonitrile (5d)*: mp: 247–249 °C (lit.^48^ 247–249 °C); Yield: 89%; ^1^H NMR (400 MHz, DMSO-*d*_*6*_): δ 5.62 (s, 2H, NH_2_), 7.34–7.67 (m, 4H, CH_Aro_), 8.63 (s, 2H, NH_2_) ppm; ^13^C NMR (100 Hz, DMSO-*d*_6_): δ = 87.7, 116.2, 116.4, 122.2, 124.6, 130.1, 133.1, 133.2, 158.0, 160.5, 163.1, 164.1 ppm.

*1,6-diamino-4-(2-nitrophenyl)-2-oxo-1,2-dihydropyridine-3,5-dicarbonitrile (5e)*: m.p: 234–235 °C (lit.^48^ 234–236); Yield: 90%; ^1^H NMR (400 MHz, DMSO-*d*_*6*_): δ 5.54 (s, 2H, NH_2_), 7.55–7.89 (m, 4H, CH_Aro_), 8.21 (s, 2H, NH_2_) ppm; ^13^C NMR (100 Hz, DMSO-*d*_6_): δ 87.2, 98.0, 113.7, 114.3, 119.0, 125.0, 126.4, 129.4, 132.1, 134.3, 149.3, 155.3, 158.7 ppm.

*1,6-diamino-2-oxo-4-phenyl-1,2-dihydropyridine-3,5-dicarbonitrile (5f)*: m.p = 238–240 °C (lit.^47^ 237–240); Yield: 87%; ^1^H NMR (400 MHz, DMSO-*d*_*6*_): δ 5.69 (s, 2H, NH_2_), 7.51–7.63 (m, 5H, CH_Aro_), 8.51 (s, 2H, NH_2_) ppm; ^13^C NMR (100 Hz, DMSO-*d*_6_): δ 74.3, 86.4, 115.4, 116.3, 128.0, 128.6, 130.2, 134.6, 156.6, 159.2, 159.5.

*1,6-diamino-4-(4-(dimethylamino)phenyl)-2-oxo-1,2-dihydropyridine-3,5-dicarbonitrile (5g)*: m.p = 248–250 °C (lit.^47^ 249–251); Yield: 99%; ^1^H NMR (400 MHz, DMSO-*d*_*6*_*,* ppm) δ: 3.07 (s, 6H, 2CH_3_), 5.67 (s, 2H, NH_2_) ppm, 6.86 (d, 2H, *J* = 8.8 Hz, CH_Aro_), 7.41 (d, 2H, *J* = 8.4 Hz, CH_Aro_), 8.38 (brs, 2H, NH_2_) ppm; ^13^C NMR (100 Hz, DMSO-*d*_6_): δ = 73.6, 85.5, 111.1, 111.7, 116.2, 120.6, 128.9, 129.5, 151.5, 156.7, 159.5, 159.6 ppm.

*1,6-diamino-4-(4-hydroxyphenyl)-2-oxo-1,2-dihydropyridine-3,5-dicarbonitrile (5h)*: m.p: 324–326 °C (lit.^47^ 325–327 °C); Yield: 99%,; ^1^H NMR (400 MHz, DMSO-*d*_*6*_) δ: 5.64 (s, 2H, NH_2_), 6.90 (d, 1H, *J* = 8.4 Hz, CH_Aro_), 6.95 (d, 1H, *J* = 8.8 Hz, CH_Aro_), 7.34 (d, 1H, *J* = 8.4 Hz, CH_Aro_), 8.02 (d, *J* = 8.4 Hz, 1H, CH_Aro_), 8.40 (brs, 1H, NH_2_), 10.05 (s, 1H, OH) ppm; ^13^C NMR (100 Hz, DMSO-*d*_6_): δ 74.1, 115.2, 116.4, 122.5, 124.8, 129.9, 134.0, 154.8, 156.6, 159.3, 162.9 ppm.

*1,6-diamino-4-(3-nitrophenyl)-2-oxo-1,2-dihydropyridine-3,5-dicarbonitrile (5i)*: m.p: 250–251 °C (lit.^49^ 249–251 °C); Yield 98%; ^1^H NMR (400 MHz, DMSO-*d*_*6*_) δ: 5.68 (s, 2H, N-NH_2_), 6.42 (brs, 2H, H-Ar), 6.66 (brs, 2H, H-Ar), 8.83 (brs, 2H, NH_2_) ppm. ^13^C NMR (100 Hz, DMSO-*d*_6_): δ 76.2, 91.4, 116.7, 125.8, 130.6, 134.4, 135.2, 148.1, 160.3, 160.5, 160.6 ppm.

*1,6-diamino-4-(2,4-dimethoxyphenyl)-2-oxo-1,2-dihydropyridine-3,5-dicarbonitrile (5j)*: m.p: 252–253 °C (lit.^42^ 251–253); Yield: 99%, ^1^H NMR (400 MHz, DMSO-*d*_*6*_): δ 3.62 (s, 3H, OCH_3_), 3.85 (s, 3H, OCH_3_), 5.51 (S, 2H, NH_2_), 6.66–7.24 (m, 3H, CH_Aro_), 8.85 (s, 2H, NH_2_) ppm; ^13^C NMR (100 Hz, DMSO-*d*_6_): δ 55.1, 55.5, 66.1, 105.0, 118.9, 119.4, 130.7, 132.0, 159.3, 161.4, 162.2, 164.9, 174.6 ppm.

*1,6-diamino-2-oxo-4-(p-tolyl)-1,2-dihydropyridine-3,5-dicarbonitrile (5k)*: m.p: 238–240 °C (lit.^47^ 240–241 °C); Yield: 97%; ^1^H NMR (400 MHz, DMSO-*d*_*6*_): δ 5.66 (s, 2H, NH_2_), 7.35–7.40 (m, 4H, CH_Aro_), 8.45 (brs, 2H, NH_2_) ppm. ^13^C NMR (100 Hz, DMSO-*d*_6_): δ 20.9, 74.2, 86.3, 115.5, 116.4, 127.9, 129.1, 131.6, 140.0, 156.6, 159.3, 159.6 ppm.

*1,6-diamino-4-(furan-2-yl)-2-oxo-1,2-dihydropyridine-3,5-dicarbonitrile (5l)*: m.p: 323–326 °C (lit.^47^ 325–327 °C); Yield: 98%; ^1^H NMR (400 MHz, DMSO-*d*_*6*_*,*) δ: 5.79 (s, 2H, NH_2_), 7.49–7.62 (m, 3H, CH_Aro_), 8.17 (s, 2H, NH_2_) ppm; ^13^C NMR (100 Hz, DMSO-*d*_6_): δ = 80.0, 114.4, 137.4, 139.2, 143.5, 146.5, 148.2, 150.2, 155.9, 164.1, 171.6 ppm.

*1',6'-diamino-2'-oxo-1',2'-dihydro-[2,4'-bipyridine]-3',5'-dicarbonitrile (5m)*: m.p: 296–297 °C; Yield: 90%; ^1^H NMR (400 MHz, DMSO-*d*_*6*_): δ 5.79 (s, 2H, NH_2_), 7.64–7.88 (m, 4H, CH_Aro_), 8.81 (s, 2H, NH_2_) ppm; ^13^C NMR (100 Hz, DMSO-*d*_6_): δ 85.2, 113.9, 121.8, 123.7, 129.6, 134.7, 140.9, 156.5, 161.6, 183.7, 187.7, 188.2 ppm.

## Results and discussion

After preparing metal–organic framework nanostructures (Fe-MOF) via co-precipitation method using Fe (NO_3_)_3_∙9H_2_O as iron precursor and 1,3,5-benzenetricarboxylic acid as organic ligand CuO nanostructures were added to modify the surface of metal–organic nano-framework. The mentioned nanostructures were placed on the metal–organic nano-framework by co-precipitation method to achieve Rod-like trifunctional Fe-based MOF@CuO nanocomposites (RL TF Fe-based MOF@CuO NC) as nano-catalyst.

### Characterization of RL BF Fe-based MOF@CuO NC

Different procedures, including XRD, FE-SEM, EDX, TGA, BET, and VSM, were used to characterize RL TF Fe-based MOF@CuO NC.

**Figure 1:**
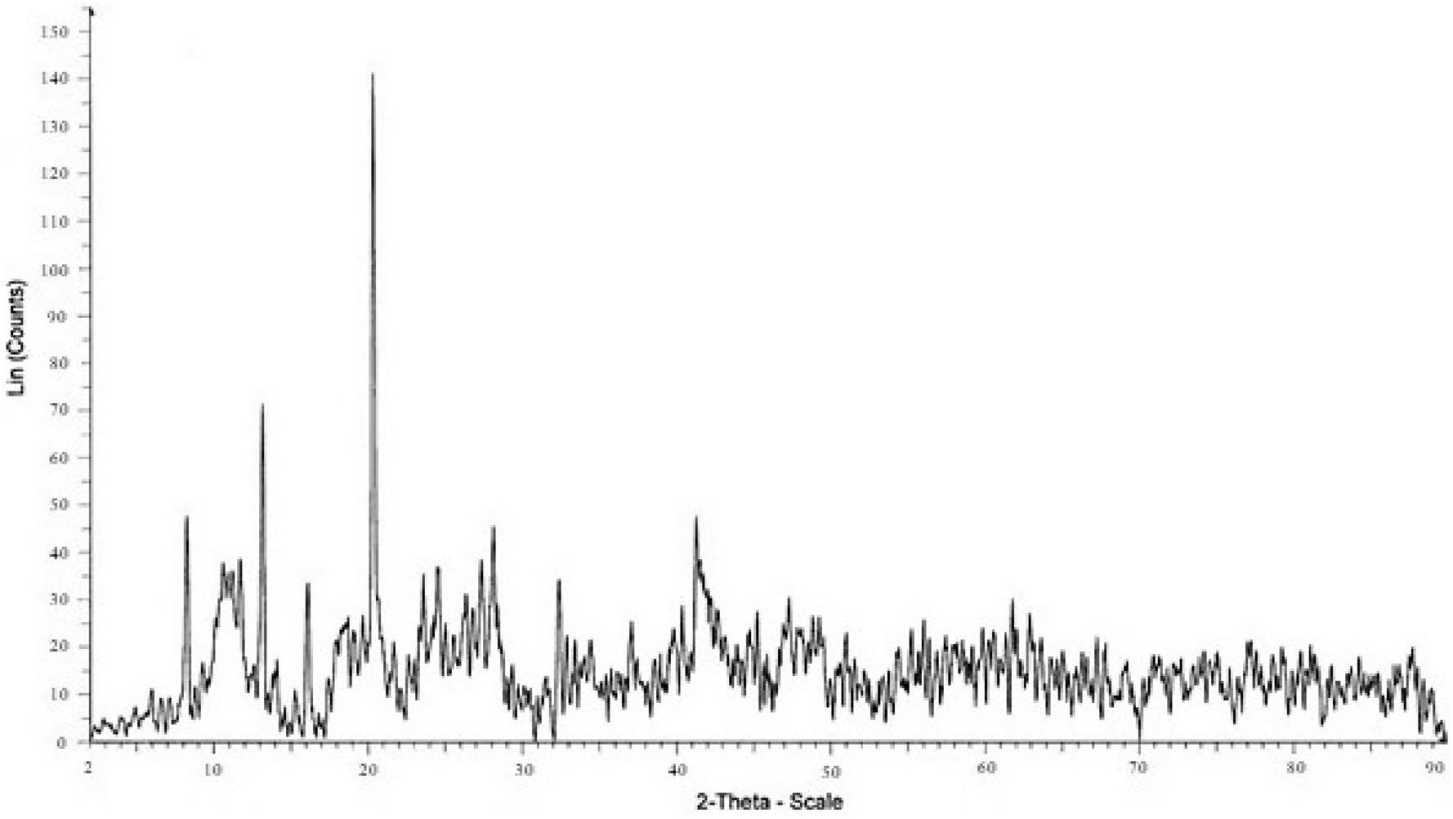
XRD pattern of RL BF Fe-based MOF@CuO NC.

The XRD pattern of the RL TF Fe-based MOF@CuO NC is shown in Fig. 1. Accordingly, the CuO diffraction peaks are covered by TF Fe-based MOF diffraction peaks. Debye–Scherrer formula (Eq. 1) was employed to estimate the crystallite size of the obtained RL TF Fe-based MOF@CuO NC.

1 $${\text{D }} = \frac{{0.9{\uplambda }}}{\beta cos\theta }$$

In which, λ, θ, and β represent the X-ray wavelength (1.54056 Å for Cu lamp), half of the Bragg diffraction angle, and half of the width of maximum intensity diffraction peak, respectively. RL TF Fe-based MOF@CuO NC had a mean crystallite size of 83.4 nm.

**Figure 2:**
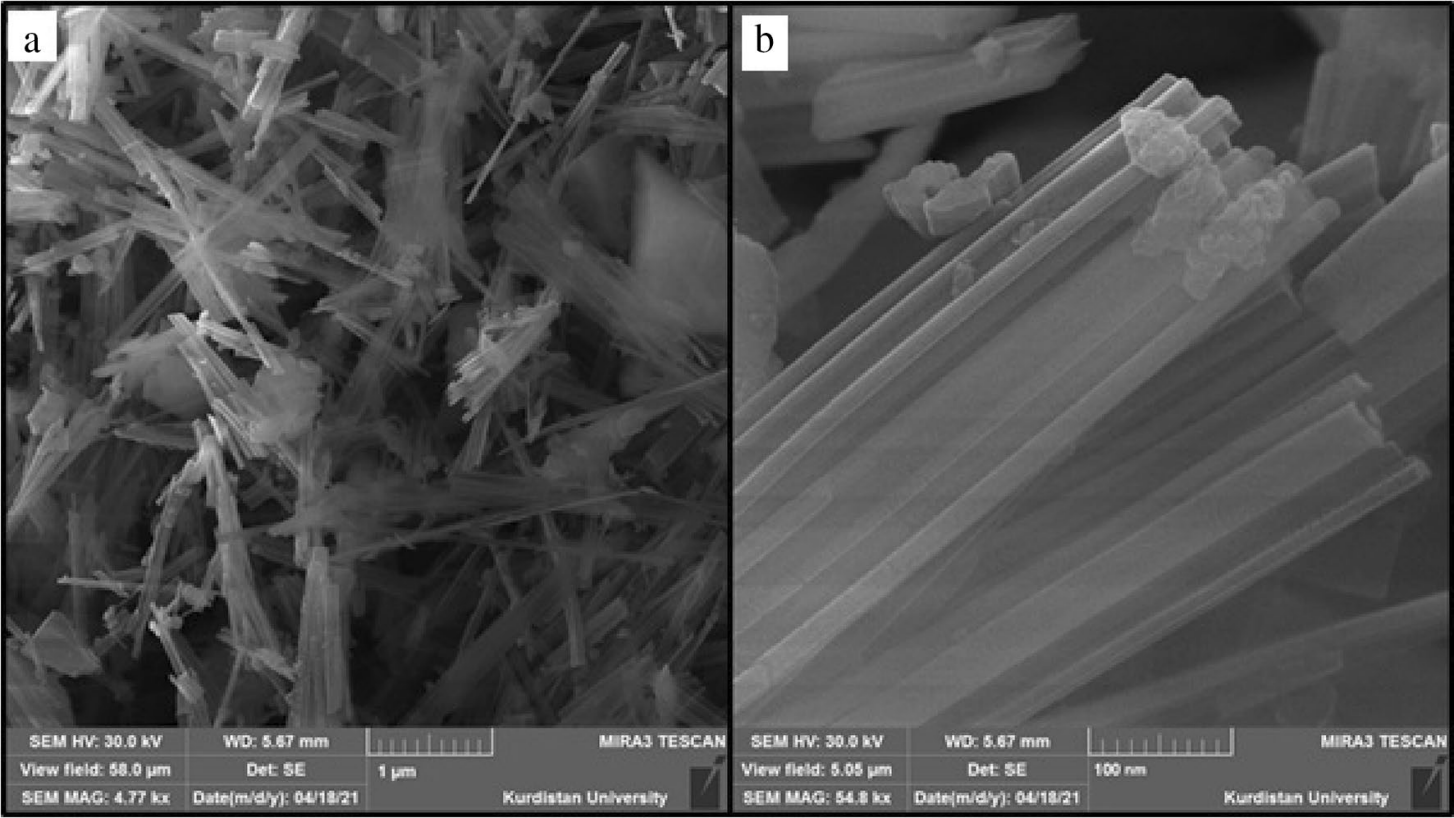
(**a**) SEM image and (**b**) high-resolution SEM image of RL BF Fe-based MOF@CuO NC.

Figure 2a indicates a low-magnification FE-SEM image of the produced RL TF Fe-based MOF@CuO NC. The TF Fe-based MOF@CuO NC showed a rod-like structure. Based on Fig. 2b, the rods have a length and diameter of nearly 1–3 μm and 10–30 nm, respectively. Uniform coverage of the study area by the nanorods was evident. Morphologically, the RL TF Fe-based MOF@CuO NC showed a straight rod characteristic, as well as a smooth surface (Fig. 2b).

**Figure 3:**
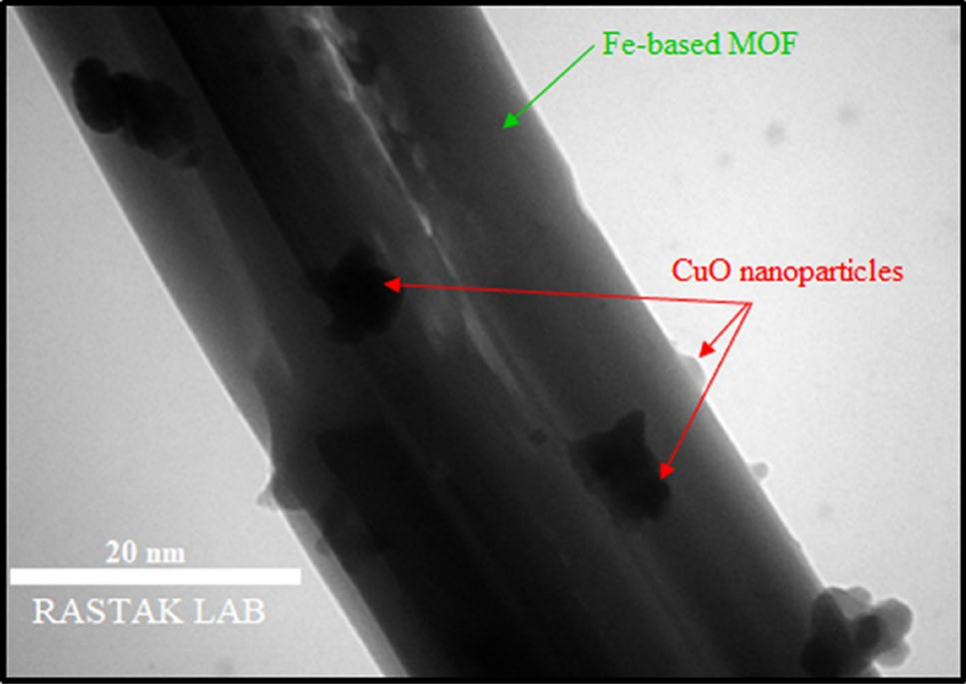
TEM image of RL BF Fe-based MOF@CuO NC.

Figure 3 shows a TEM image of RL BF Fe-based MOF@CuO NC. It can be observed that the diameter of Fe-based MOF nanorods is about 30 nm. The surface of Fe-based MOF nanorods is homogeneously covered with well-dispersed spherical CuO nanoparticles. The particle size of the CuO nanoparticles is different. It can be found that the diameter of CuO nanoparticles is mostly distributed between 2 and 7 nm.

**Figure 4:**
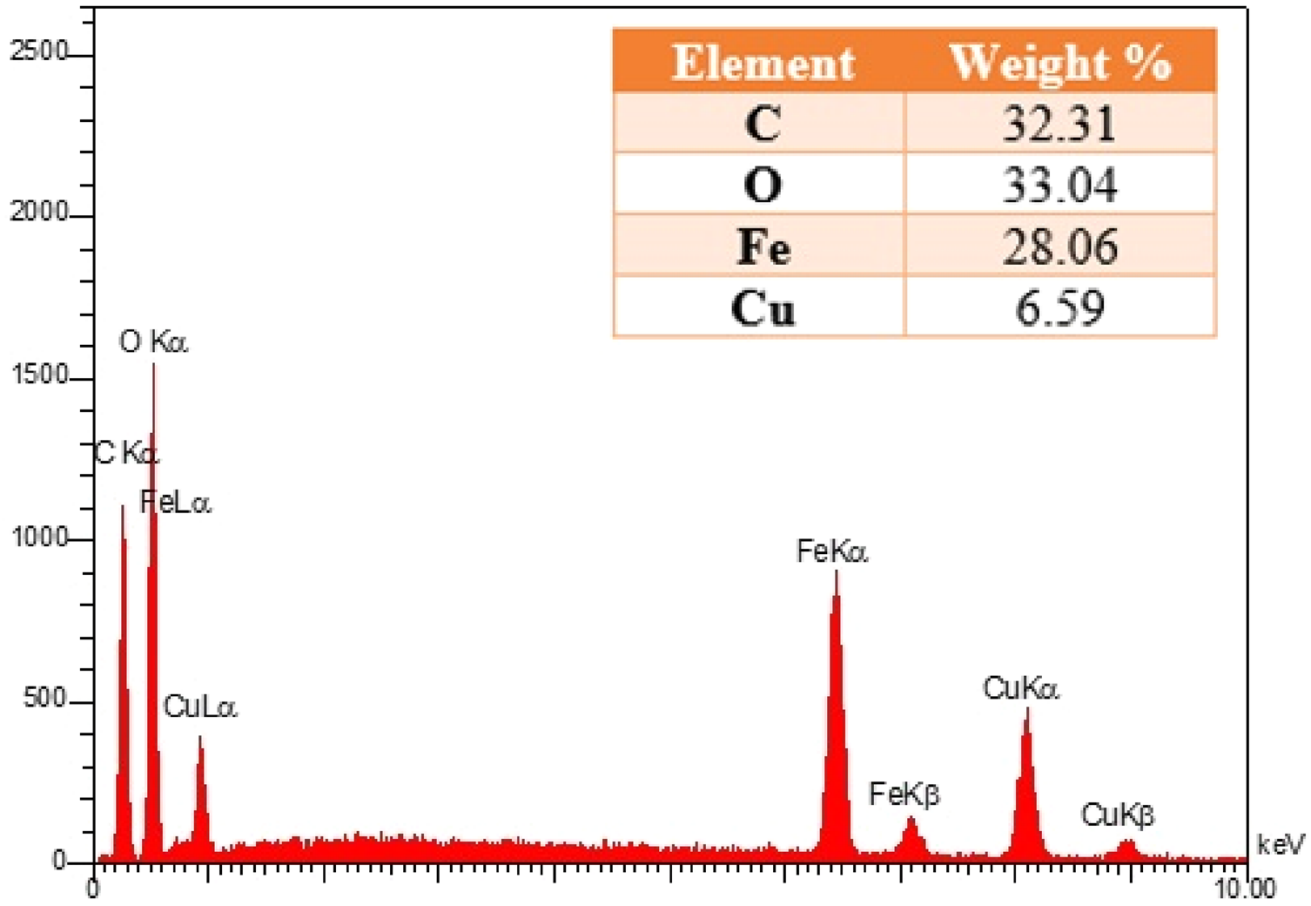
EDX spectra of RL BF Fe-based MOF@CuO NC.

Figure 4 shows EDX results of RL TF Fe-based MOF@CuO NC. The RL TF Fe-based MOF@CuO NC, contain C, O, Cu, and Fe with the atomic ratio of C: O: Cu: Fe ≈ 33.14: 12.55: 3.76: 50.55, with no other impurity peaks.

**Figure 5:**
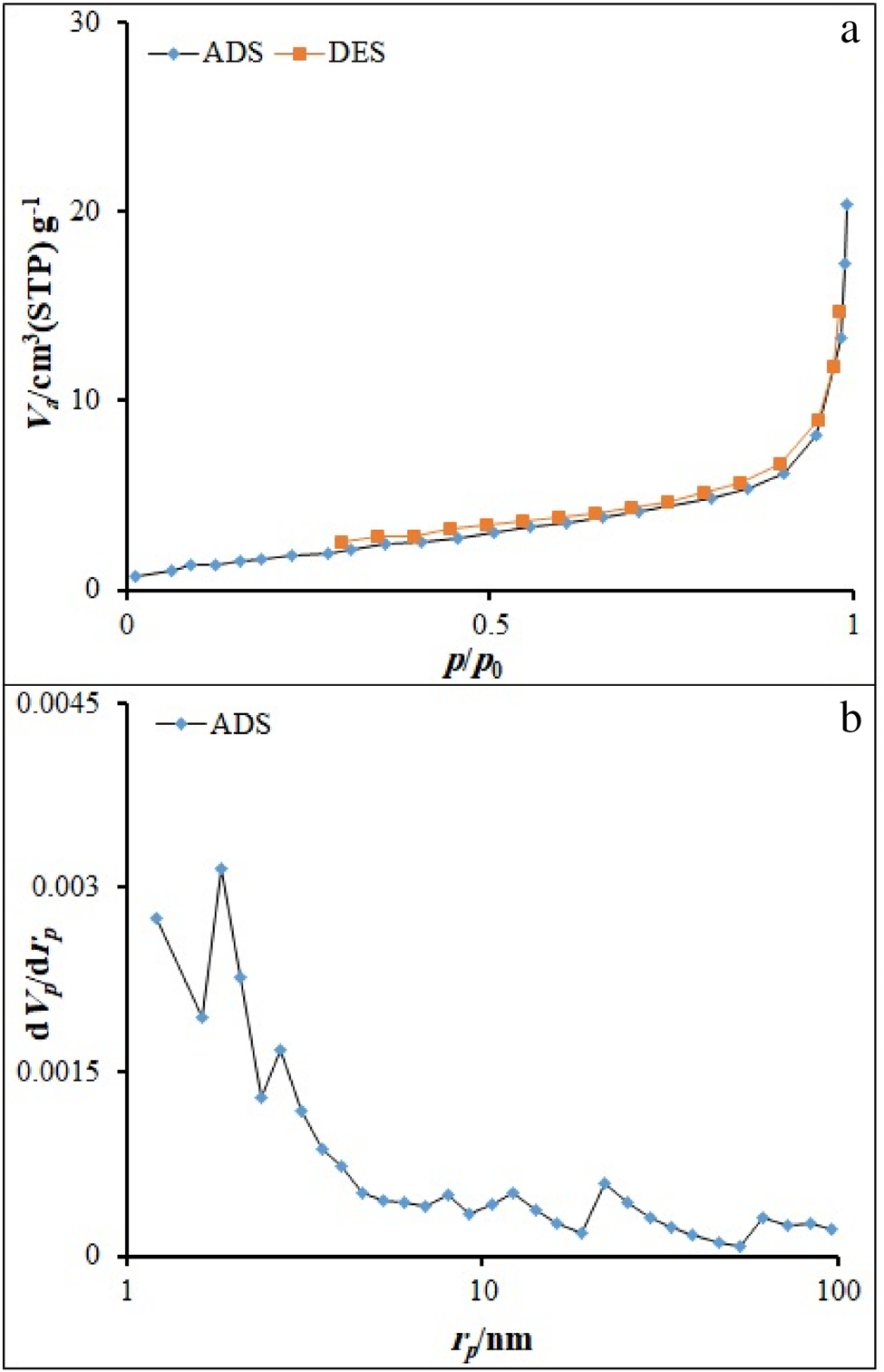
(**a**) N_2_ adsorption–desorption isotherms RL BF Fe-based MOF@CuO NC and (**b**) BJH results obtained for RL BF Fe-based MOF@CuO NC.

Figure 5 indicates the N_2_ adsorption–desorption isotherm of the RL TF Fe-based MOF@CuO NC. Physical characteristics, such as the surface area, pore size, and volume, and distribution, were determined through N_2_ sorption estimations. Type IV isotherm can be recognized in the case of RL TF Fe-based MOF@CuO NC with a distinct hysteresis loop. The surface area, the pore volume, and pore size of RL TF Fe-based MOF@CuO NC were 8.4228 m^2^ g^−1^, 0.031269 cm^3^ g^−1^, and 17.346 nm, respectively.

**Figure 6:**
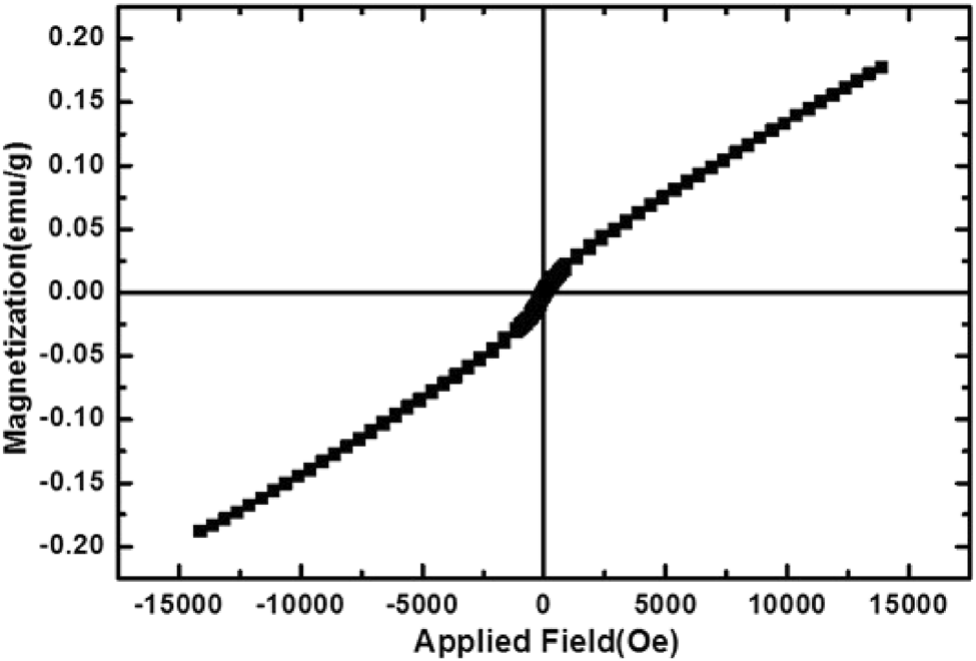
VSM magnetization curves of RL BF Fe-based MOF@CuO NC.

The VSM analysis was utilized at room temperature to characterize the magnetic properties of the RL TF Fe-based MOF@CuO NC. Figure 6 presents the M–H curve of the nanocomposite at room temperature. Accordingly, the semiconductor materials show magnetic characteristics with strong dependence on structural, morphological, and crystal geometric factors. Typical M–H curves represent a weak paramagnetic due to limited spin orientation at the maximum applied field (0.012 Oe). Nanocomposite has a coercivity of Hc, 75.0 Oe, suggesting their magnetic behavior with saturation magnetism of Ms, 0.17 emu. g^−1^.

**Figure 7:**
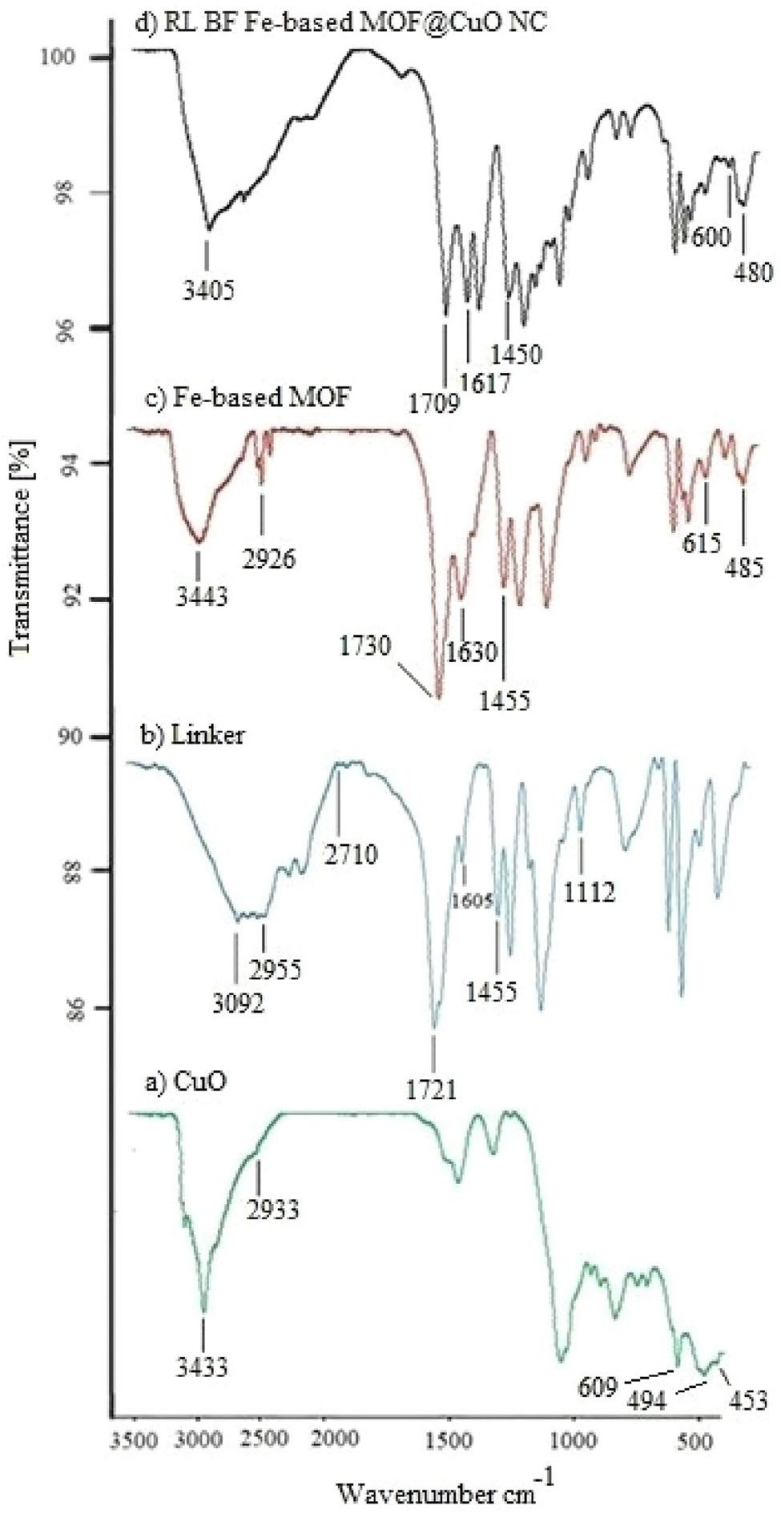
IR (KBr, υ cm^−1^) curve of (**a**) CuO, (**b**) 1,3,5-benzenetricarboxylic acid linker, (**c**) synthesized Fe-based MOF and (**d**) RL BF Fe-based MOF@CuO NC.

The FT-IR spectra of CuO, TF Fe-based MOF, and RL TF Fe-based MOF@CuO NC are shown in Fig. 7. FTIR spectroscopy can be employed for identifying molecule functional groups as each chemical bond has a distinct energy absorption band, through which the structural and bond information of compounds can be obtained. The FTIR spectrum related to the recently obtained cupric oxide sample is shown in Fig. 7a. The peaks observed at 453, 494, 609 cm^−1^ represent the Cu–O bond characteristic stretching vibration of CuO, which agrees with literature values. The Sharp peak at 609 cm^-1^ for CuO nanoparticles and the wide peak at 3433 cm^−1^ represent Cu–O bond formation and O–H stretching of the moisture content, respectively^53^.

Water attachment to the surface of CuO nanoparticles can justify this phenomenon, while it is possible to remove this reaction byproduct through additional heating. The O–H bond symmetric and asymmetric stretching vibrations are manifested by the respective bands at 2933 and 3433 cm^−1^.

The IR spectrum of 6c shows that the addition of iron nitrate to the linker and formation Fe-MOF led to a rise in the absorption of carbonyl group due to the coordination of Fe metal with acidic OH. Also, according to the IR spectrum of 6d addition of of Fe-MOF to the CuO nanoparticles and the formation of Fe-MOF@CuO decreased the absorption of carbonyl due to the coordination of the CuO nanoparticle with the oxygen of the carbonyl group.

Thermal analysis of a representative sample of the synthesized compound, RL BF Fe-based MOF@CuO NC, is shown in Table 1. The weight losses started at 50 °C and ended at 500 °C, approximately. The 1st and 2st temperatures are 67 °C and 92 °C, where minor weight losses result from the vanished solvent and evaporated trapping solvent, respectively. Proportionate to temperature ascent from 273 to 315 °C was observed. That probably corresponds to decomposing linker on the skeleton. The weight loss of dissociation of coordinated water for nanocomposite was estimated in the range of 420–462 °C. Therefore, obtained data show high thermal stability in elevated temperatures.

**Table 1:** TGA data of RL BF Fe-based MOF@CuO NC.

Steps no	Temperature (°C)	Results
I	67	Vanished solvent
II	92	Evaporated trapping solvent
III	273–315	Ligand decomposition
IV	420–462	Decomposition of coordinated water

### Synhtesis of N‑amino-2-pyridones in the presence of RL BF Fe-based MOF@CuO NC as catalyst

**Figure 8:**
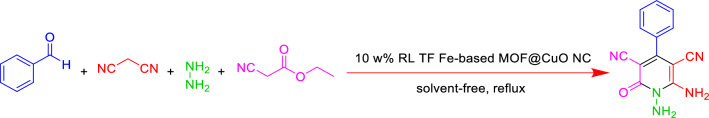
Four-component synthesis of N‑amino-2-pyridones catalyzed by RL BF Fe-based MOF@CuO NC under solvent-free and reflux conditions.

After successful preparation and characterization of nano organo-catalyst by SEM, EDS, mapping, XRD, BET, TGA and VSM, it was applied in prominent organic reactions to prepare N-amino-2-pyridones through one-pot four-component reaction among methyl cyanoacetate, Hydrazine hydrate, malononitrile and aromatic aldehydes to examine the catalytic function of RL BF Fe-based MOF@CuO NC as effective Lewis acid catalyst (Fig. 8).

The effects of various parameters were initially studied by testing a model reaction of methyl cyanoacetate (1 mmol), Hydrazine hydrate (1 mmol), malononitrile (1 mmol) and 4-methoxybenzaldehyde (1 mmol) to achieve optimal reaction conditions. Investigations were primarily performed to determine the contribution of the solvent to the reactions through the model reaction. Hence, this step was carried out using different solvents, including water, ethanol, methanol, acetonitrile and *n*-hexane solvents. Besides, solvent-free conditions were also examined in the presence of 10 w% of catalytic of RL TF Fe-based MOF@CuO NC at reflux conditions. According to the results, the solvent-free condition was the most appropriate to prepare Namino-2-pyridones, and the reaction time gradually decreased by using highly polar to less polar solvents (Table 2).

**Table 2:** Optimization of the reaction conditions for the synthesis of N‑amino-2-pyridones derivatives using RL BF Fe-based MOF@CuO NC.

Entry	Catalyst	Solvent	Tem (°C)	Time (min h^−1^)	Yield (%)^b^
1	RL BF Fe-based MOF@CuO NC 10 w%	H_2_O	Reflux	15 min	88
2	RL BF Fe-based MOF@CuO NC 10 w%	C_2_H_5_OH	Reflux	23 min	72
3	RL BF Fe-based MOF@CuO NC 10 w%	CH_3_OH	Reflux	30 min	64
4	RL BF Fe-based MOF@CuO NC 10 w%	CH_3_CN	Reflux	3 h	40
5	RL BF Fe-based MOF@CuO NC 10 w%	n-Hexane	Reflux	4 h	23
6	RL BF Fe-based MOF@CuO NC 10 w%	Free-solvent	Reflux	5 min	96
7	RL BF Fe-based MOF@CuO NC 10 w%	Free-solvent	50 °C	25 min	96
8	RL BF Fe-based MOF@CuO NC 10 w%	Free-solvent	r.t.	45 min	81
9	RL BF Fe-based MOF@CuO NC 15 w%	Free-solvent	Reflux	5 min	96
10	RL BF Fe-based MOF@CuO NC 5 w%	Free-solvent	Reflux	20 min	76
11	RL BF Fe-based MOF@CuO NC 20 w%	Free-solvent	Reflux	5 min	96
12	Free-catalyst	Free-solvent	Reflux	8 h	Trace

^a^Reaction conditions: 4-methoxybenzaldehyde (3 mmol), malononitrile (3 mmol), methyl cyanoacetate (3 mmol), Hydrazine hydrate (3 mmol) and RL BF Fe-based MOF@CuO NC (10 w%) under different conditions.

^b^Yield refer to isolated products.

In the next step, the effect of reaction temperature was tested considering room temperature, 50 and reflux conditions. Temperature decline below 50 ºC decremented the yield of the product and increased the reaction time. Moreover, increasing temperature from 50 °C to reflux conditions was improved the progress of the reaction (Table 2, entries 6–8).

The catalyst amount was optimized through the model reaction in solvent-free conditions in the absence and presence of various catalyst amounts. The yield was 76%, 98%, 98%, and 98% when using 5, 10, 15, 20 w% of RL BF Fe-based MOF@CuO NC, respectively (Table 2). The results indicate the RL BF Fe-based MOF@CuO NC catalyst contributes significantly to achieving the response so that reaction produced trace product in the absence of catalyst after prolonged reaction time while 10 w% catalyst seemed to be enough for the reaction to proceed, leading to considerably high yields while requiring shorter reaction times. Hence, a decrease in the catalyst amount resulted in the efficiency reduction, while higher catalyst amounts led to no significant efficiency improvements (Table 2, entry 6 and entries 9–12).

Under optimal reaction conditions, the scope, generality, and applicability of newly introduced protocol were checked by various aromatic aldehydes, with substituents that withdraw or donate electrons (Table 3), malononitrile, methylcyanoacetate and hydrazine hydrate were used to perform the reaction in the presence of 10 w% RL BF Fe-based MOF@CuO NC under solvent-free and reflux conditions. As Table 3 indicates, aldehydes with electron-withdrawing or electron-donating groups offer corresponding N-amino-2-pyridones with considerably high product yield (87–99%) while requiring shorter reaction times (2–5 min) with no side reactions.

**Table 3:** Preparation of N‑amino-2-pyridones in the presence of RL BF Fe-based MOF@CuO NC (10 w%) under optimized reaction conditions.

Entry^a^	R (aldehyde)	Product	Time (min)	Yield (%) ^b^	*m. p.* (°C)		TON^c^	TOF^d^
					Found	Reported [ref.]		
1	4-OCH_3_C_6_H_4_-	5a	5	98	220–222	222–224^54^	6.154	1.231
2	4-ClC_6_H_4_-	5b	3	87	340–343	341–342^54^	5.577	1.859
3	4-NO_2_C_6_H_4_-	5c	2	91	> 340	> 340^54^	5.833	2.916
4	2-FC_6_H_4_-	5d	4	89	247–249	249–251^55^	7.12	1.78
5	2-NO_2_C_6_H_4_-	5e	5	90	234–235	234–236^55^	5.769	1.154
6	C_6_H_5_-	5f.	3	87	238–239	237–240^54^	6.96	2.32
7	4-N(CH_3_)_2_C_6_H_4_-	5 g	3	99	248–250	249–251^54^	6.346	2.115
8	4-OHC_6_H_4-_	5 h	5	99	324–326	325–327^54^	7.92	1.584
9	3-NO_2_C_6_H_4_-	5i	5	98	250–251	249–251^56^	6.282	1.256
10	2,4-(OCH_3_)_2_C_6_H_3_-	5j	3	99	252–253	251–253^42^	5.294	1.765
11	4-CH_3_C_6_H_4_-	5 k	5	97	238–240	240–241^54^	7.76	1.552
12	C_5_H_4_O_2_	5 l	8	98	323–326	325–327^54^	10.538	1.317
13	C_6_H_5_NO	5 m	10	90	296–297	–	7.2	0.72

^a^Reaction conditions: methyl cyanoacetate (3 mmol), Hydrazine hydrate (3 mmol), malononitrile (3 mmol), and aryl aldehydes (3 mmol) in the presence of RL BF Fe-based MOF@CuO NC were heated under solvent-free and reflux conditions at 90 °C for appropriate times.

^b^Yield refer to isolated products.

^c^TON = (mmol of product)/(mmol of active site of catalyst).

^d^TOF (min^−1^) = TON/t (min).

To examine the advantages of this methodology, new synthesized catalyst was compared with recently introduced catalysts to synthesize N-amino-2-pyridones (Table 4). According to the results, the proposed protocol was highly efficient due to the solvent-free conditions and excellent catalytic effects within short reaction time.

**Table 4:** The comparison of the catalytic activity of RL BF Fe-based MOF@CuO NC with previously reported catalysts.

Entry	Catalyst	Amount of catalyst	Conditions	Time (min)	Yield (%)	Refs.
1	ZrP_2_O_7_ NPs	20 mol%	EtOH, Reflux	10	92	^38^
2	Bi (NO3)3·5H2O	0.04 g	EtOH, Reflux	8	95	^42^
3	MIL-101(Cr)-N(CH_2_PO_3_H_2_)_2_	5 mg	H_2_O, Reflux	20	92	^54^
4	ZnO NPs	8 mol%	EtOH, Reflux	40	92	^55^
5	DABCO	20 mol%	H_2_O/EtOH (1:1), r.t	90	96	^56^
6	KF-Al_2_O_3_	15 mol%	H_2_O/EtOH (1:1), r.t	30	96	^57^
7	MgO	0.25 g	EtOH, Reflux	40	83	^42^
8	CdZr_4_(PO_4_)_6_ NPs	0.06 mol%	EtOH, Reflux	30	93	^58^
9	polyaniline	0.1/50 w/v%	EtOH, Reflux	40	90	^44^
10	RL BF Fe-based MOF@CuO NC	10 w%	Solvent-free, reflux	5	98	This work

**Figure 9:**
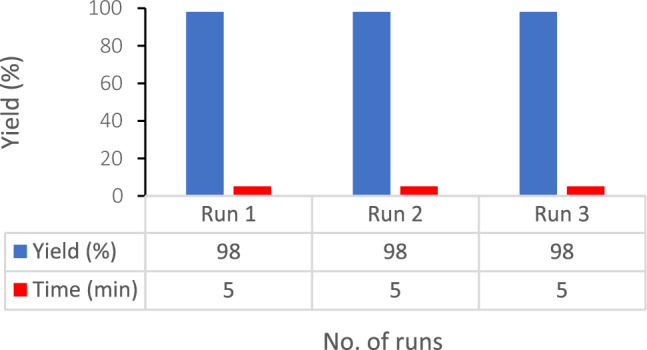
Reusability of the RL BF Fe-based MOF@CuO NC in the synthesis of 5a.

The catalyst reusability was examined through the reaction among 4-methoxybenzaldehyde (3 mmol), methyl cyanoacetate (3 mmol), hydrazine hydrate (3 mmol) and malononitrile (3 mmol), conducted as a model reaction in optimized reaction conditions. Noteworthy, the product yield was constant after recycling (run1, 98%; run 2, 98%; run 3, 98%). It is possible to reuse RL BF Fe-based MOF@CuO NC until three runs with no significant decline in its catalytic function. In the recycling procedure, hot acetone addition aimed at diluting the model reaction mixture following the reaction completion and stirring the mixture over a 5-min period. The catalyst was insoluble in acetone and underwent several washings with acetone for its separation by filtration. The above steps were followed by drying at 40 °C within an 8-h period and reusing the catalyst with no considerable decline in its catalytic effects (Fig. 9).

**Figure 10:**
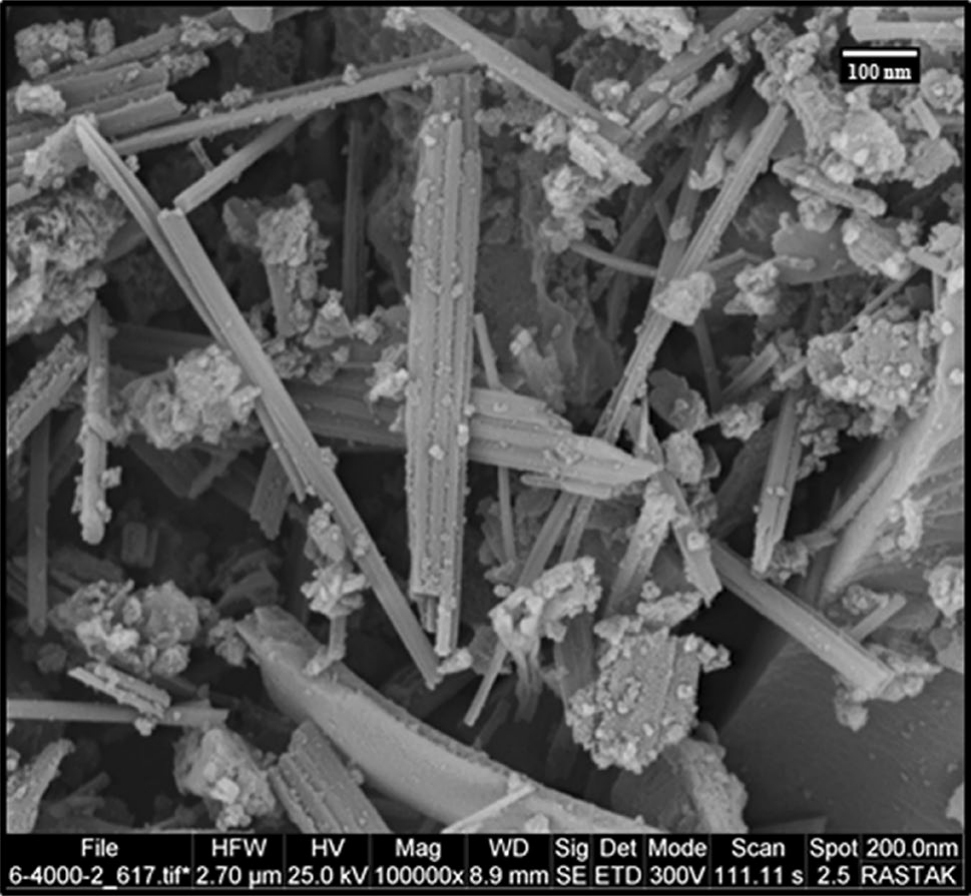
SEM images of recovered RL BF Fe-based MOF@CuO NC.

In order to indicate the stability of RL BF Fe-based MOF@CuO NC after recovering and reusing, recovered catalyst was characterized. The morphology and particle size of RL BF Fe-based MOF@CuO NC after recycling has been studied by SEM technique. The SEM image of reused RL BF Fe-based MOF@CuO NC is shown in Fig. 10. As shown in this Figure, the size and morphology of catalyst after reusing is shown a good agreement to fresh catalyst, which is about 10–30 nm in diameters. Also, the shape of the recycled catalyst was the same as the fresh catalyst, indicating its good mechanical strength.

**Figure 11:**
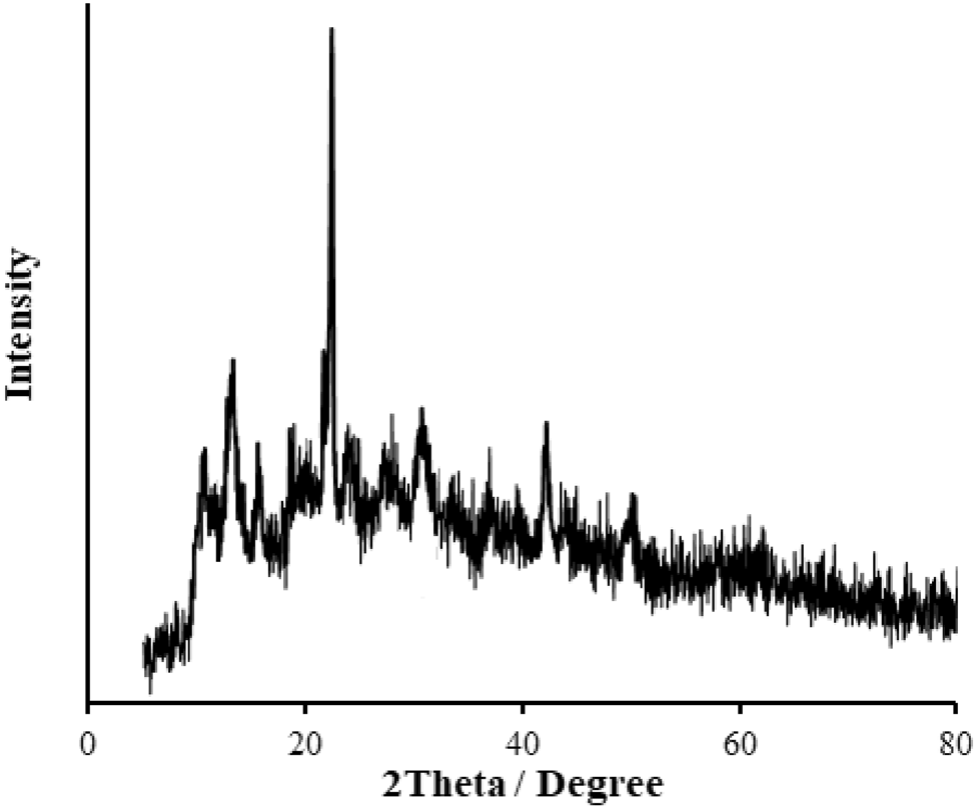
XRD Pattern of recovered RL BF Fe-based MOF@CuO NC.

The XRD of the recovered RL BF Fe-based MOF@CuO NC after the three cycle shows clear peaks corresponding to the composite at 8.43°, 13.92° and 20.57° (Fig. 11). It clearly indicates that the catalyst remains unchanged even after the reaction.

**Figure 12:**
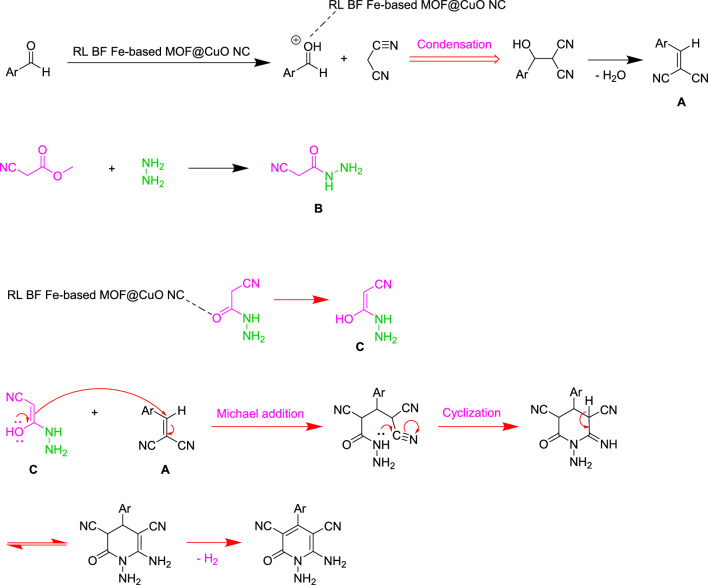
Proposed mechanism for the synthesis of N‑amino-2-pyridones.

The proposed mechanisms were used to prepare N-amino-2-pyridones in the presence of RL BF Fe-based MOF@CuO NC as shown in Fig. 12. Firstly, arylidenemalononitrile (**A**) formation was done from the Knoevenagel condensation aromatic aldehydes and malononitrile when RL BF Fe-based MOF@CuO NC was present. Meantime, methyl cyanoacetate reacts with hydrazine hydrate to form intermediate (**B)**. Then, interaction of the enolizable cyanoacetic acid hydrazide (**C**) with arylidenemalononitrile (**A**) through Michael addition, followed by booting of the intermediate’s intramolecular cyclization by nano-catalyst result in the final product yields.

## Conclusion

In summary, an effective technique was reported for the generation of useful RL TF Fe-based MOF@CuO NC nano-catalyst using 1,3,5-Benzenetricarboxylic acid linker via microwave irradiation. The catalytic activity of RL TF Fe-based MOF@CuO NC was explored in one-pot synthesis of N-amino-2-pyridones by facile, rapid and versatile multi-component reaction of malononitrile, diverse aromatic aldehydes, hydrazine hydrate and ethyl cyanoacetate under mild and solvent-free conditions. The procedure offers several advantages including environmentally friendly, excellent yields with short reaction times, simple work up procedure and the ability of recyclability catalyst for three times with no considerable functional loss.

### Supplementary Information

Supplementary Information.

## Data Availability

All data generated or analysed during this study are included in this article (and its Supplementary information files).
