# Supplementary material for: Synthesis of pyridone derivatives using 2D rod like bifunctional Fe based MOF and CuO nanocomposites as a novel heterogeneous catalyst
Source: Sci Rep. 2023 Sep 21;13:15753. doi: 10.1038/s41598-023-43045-6 (PMC10514299; doi:10.1038/s41598-023-43045-6)

***Supplementary Material***

**Synthesis of pyridone derivatives using 2D rod like bifunctional Fe based MOF and CuO nanocomposites as a novel heterogeneous catalyst**

**Negar Hoot ^1^, Enayatollah Sheikhhosseini ^1^*, Sayed Ali Ahmadi ^1^, Mahdieh Ghazizadeh ^1^, Moslem Malekshahi ^1^, Mahdieh Yahyazadehfar ^1^**

**^1^ Department of Chemistry, Kerman Branch, Islamic Azad University, Kerman, Iran**

*** Correspondence:**

Enayatollah Sheikhhosseini
sheikhhosseiny@gmail.com or sheikhhosseini@iauk.ac.ir

**^1^H NMR and ^13^C NMR of compound (5a)**

*1,6-diamino-4-(4-methoxyphenyl)-2-oxo-1,2-dihydropyridine-3,5-dicarbonitrile (****5a****)*: m.p: 220-222 °C (lit.^47^ 222-224 °C); Yield: 98 %; ^1^H NMR (400 MHz, DMSO-*d_6_*): δ 3.62 (s, 3H, OCH_3_), 3.85 (s, 3H, OCH_3_), 5.51 (s, 2H, NH_2_), 6.66-7.24 (m, 3H, CH_Ar_), 8.85 (s, 2H, NH_2_) ppm; ^13^C NMR (100 Hz, DMSO-*d*_6_): δ 55.1, 55.5, 66.1, 105.0, 118.9, 119.4, 130.7, 132.0, 159.3, 161.4, 162.2, 164.9, 174.6 ppm.

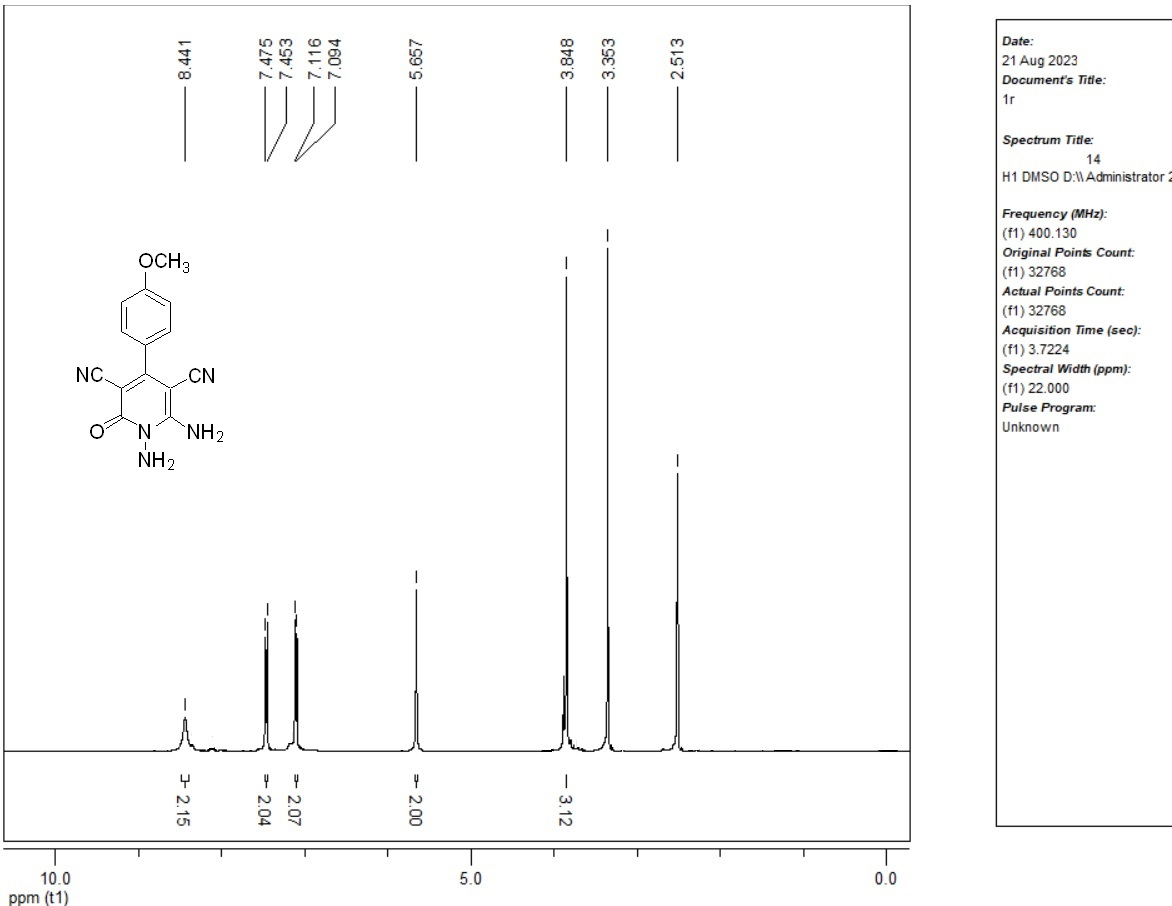


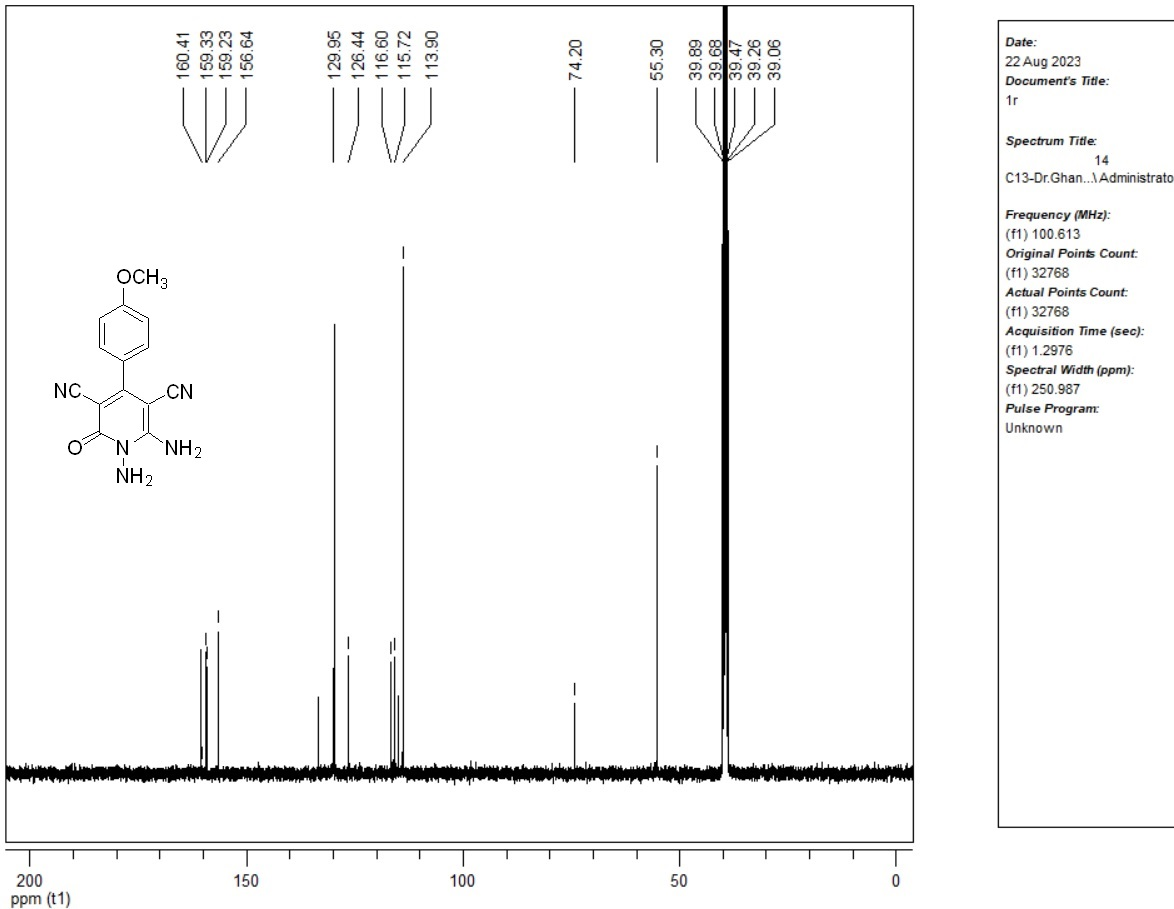


**^1^H NMR and ^13^C NMR of compound (5b)**

*1,6-diamino-4-(4-chlorophenyl)-2-oxo-1,2-dihydropyridine-3,5-dicarbonitrile (****5b****)*: m.p: 340-343 °C (lit.^47^ 341-342 °C); Yield: 87%; ^1^H NMR (400 MHz, DMSO-*d_6_*) δ : 5.71 (s, 2H, NH_2_), 7.60 (d, *J* = 7.6 Hz, 2H, CH_Aro_), 7.92 (d, *J*= 7.6 Hz, 2H, CH_Aro_), 8.74 (s, 2H, NH_2_) ppm; ^13^C NMR (100 Hz, DMSO-*d*_6_): δ = 68.7, 80.6, 115.2, 116.4, 128.3, 129.1, 130.0, 132.6, 136.0, 153.8, 158.2, 160.7 ppm.

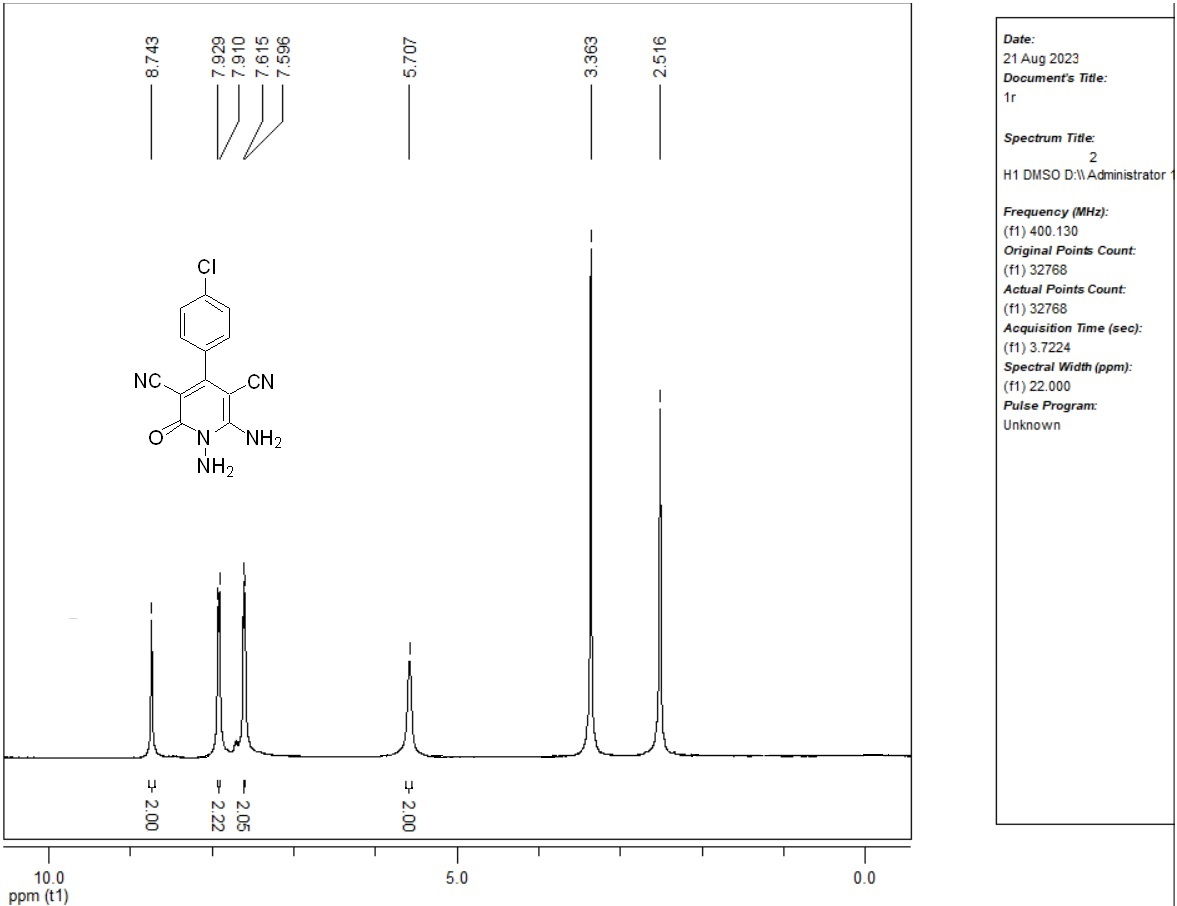


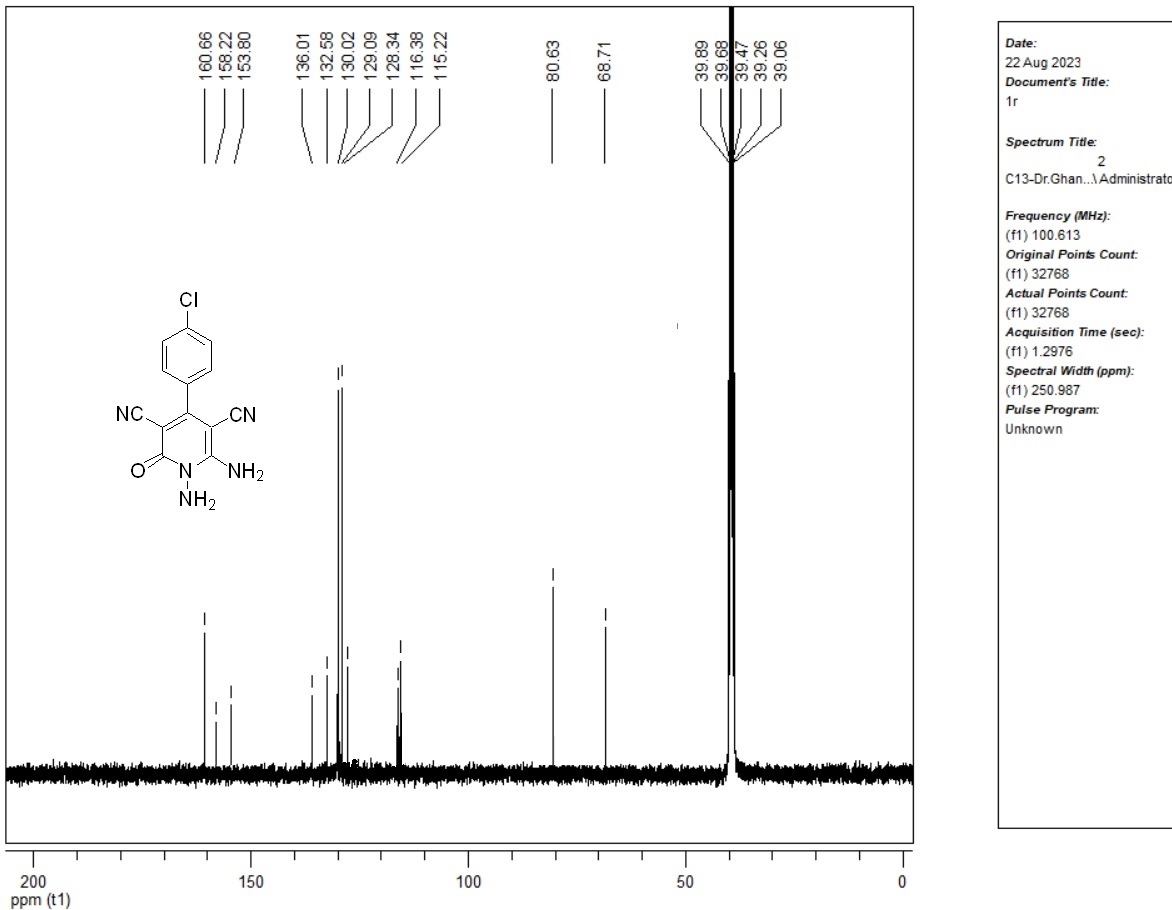


**^1^H NMR and ^13^C NMR of compound (5c)**

*1,6-diamino-4-(4-nitrophenyl)-2-oxo-1,2-dihydropyridine-3,5-dicarbonitrile (****5c****)*: m.p: > 340 °C (lit.^47^ > 340 °C); Yield: 91%; ^1^H NMR (400 MHz, DMSO-*d_6_*): δ 5.79 (brs, 2H, NH_2_), 7.63-8.44 (m, 4H, CH_Aro_), 8.75 (brs, 2H, NH_2_) ppm; ^13^C NMR (100 Hz, DMSO-*d*_6_): δ 53.6, 106.4, 114.9, 124.4, 131.7, 137.2, 149.2, 152.8, 153.3, 161.7 ppm.

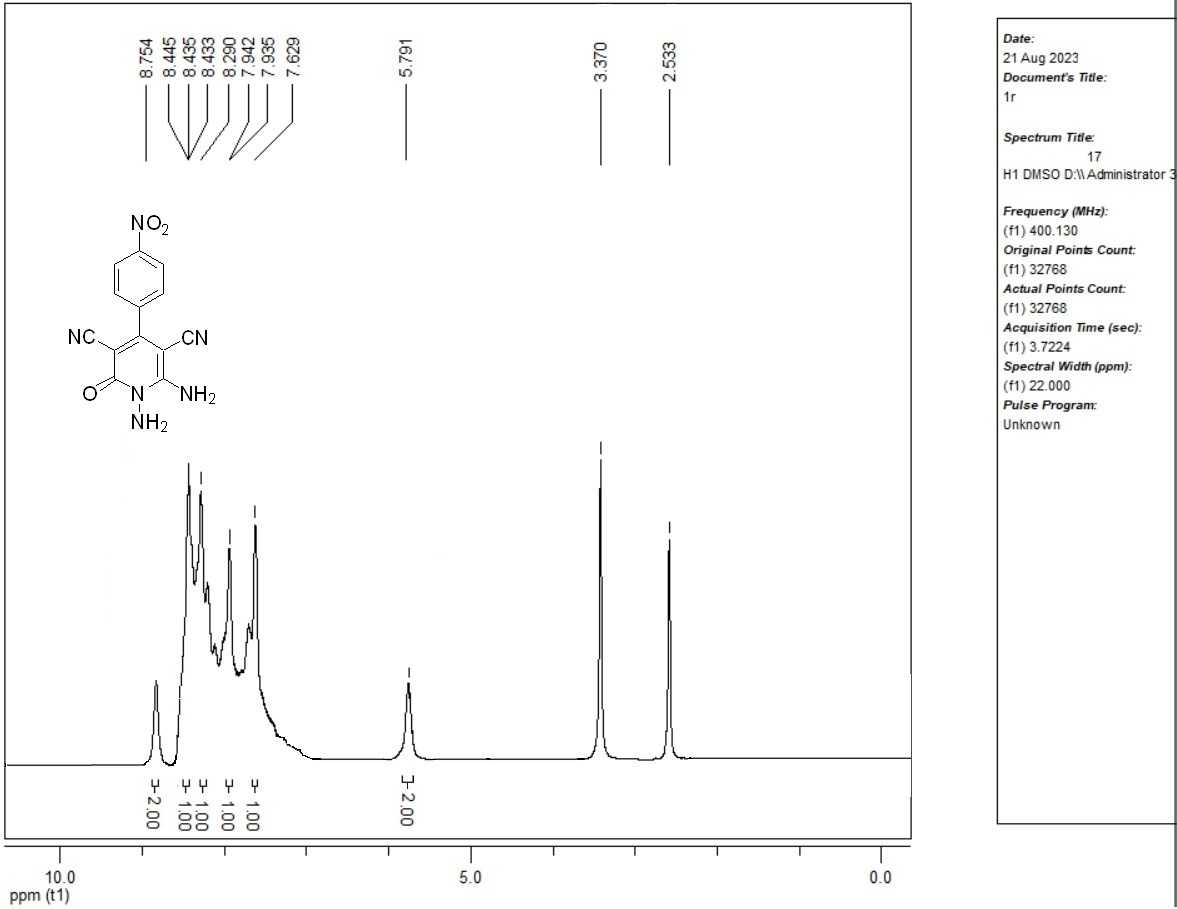


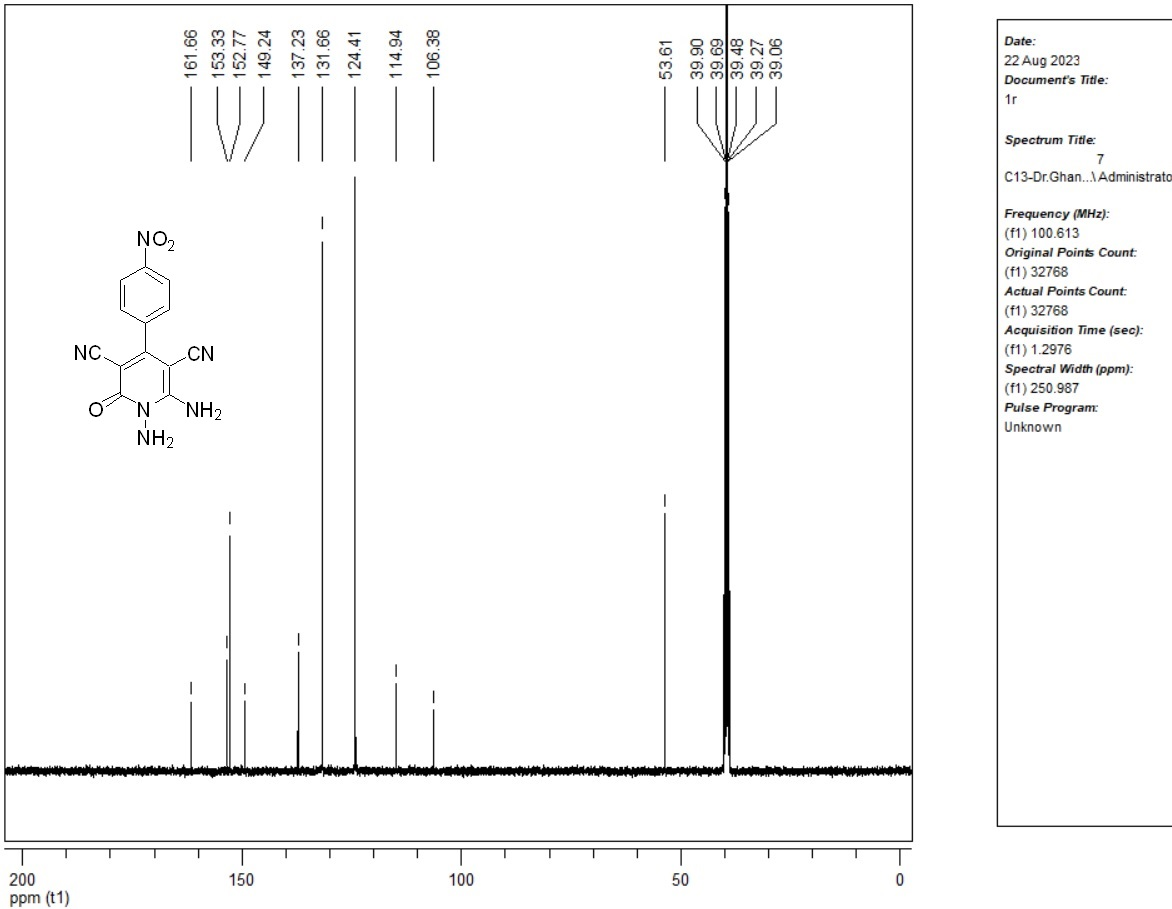


**^1^H NMR and ^13^C NMR of compound (5d)**

*1,6-diamino-4-(2-fluorophenyl)-2-oxo-1,2-dihydropyridine-3,5-dicarbonitrile (****5d****)*: mp: 247-249 °C (lit.^48^ 247-249 °C); Yield: 89%; ^1^H NMR (400 MHz, DMSO-*d_6_*): δ 5.62 (s, 2H, NH_2_), 7.34-7.67 (m, 4H, CH_Aro_), 8.63 (s, 2H, NH_2_) ppm; ^13^C NMR (100 Hz, DMSO-*d*_6_): δ = 87.7, 116.2, 116.4, 122.2, 124.6, 130.1, 133.1, 133.2, 158.0, 160.5, 163.1, 164.1 ppm.

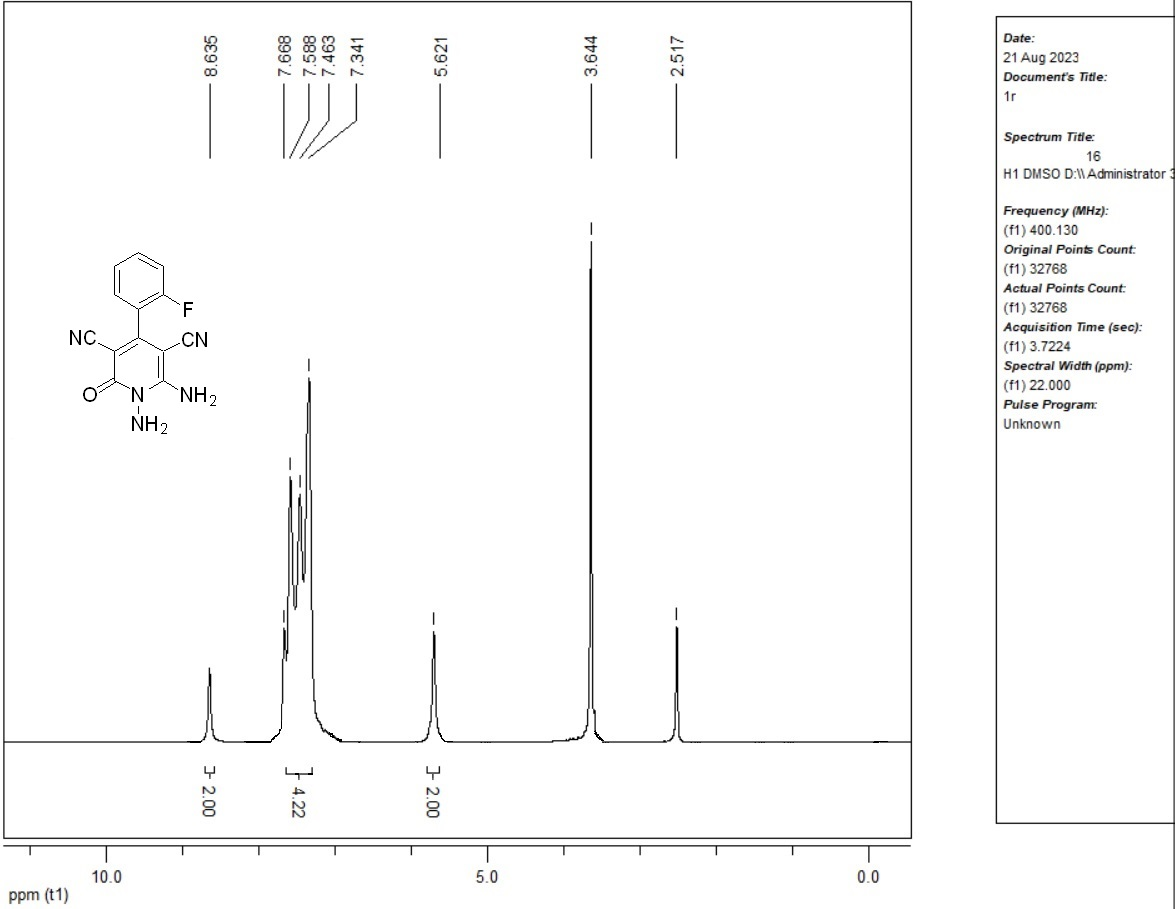


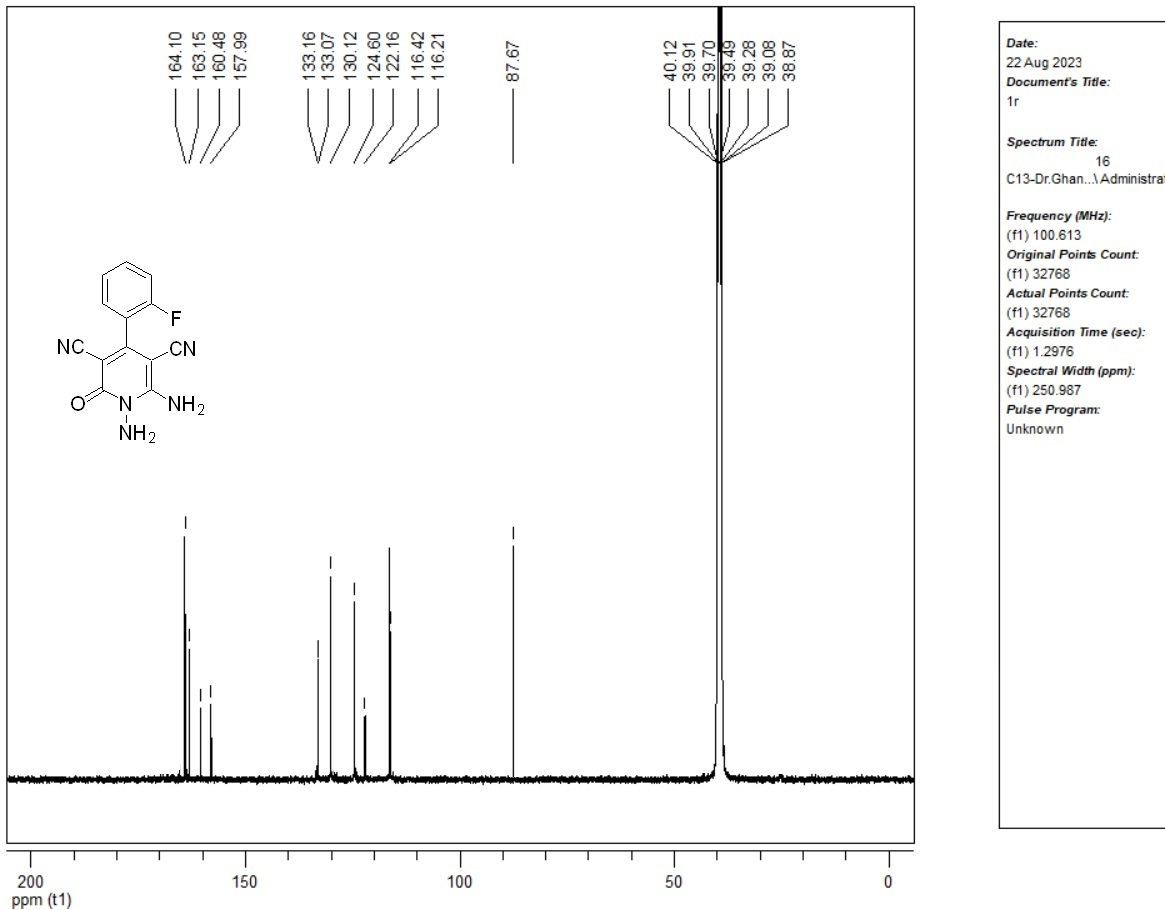


**^1^H NMR and ^13^C NMR of compound (5e)**

*1,6-diamino-4-(2-nitrophenyl)-2-oxo-1,2-dihydropyridine-3,5-dicarbonitrile (****5e****)*: m.p: 234-235 °C (lit.^48^ 234-236); Yield: 90%; ^1^H NMR (400 MHz, DMSO-*d_6_*): δ 5.54 (s, 2H, NH_2_), 7.55-7.89 (m, 4H, CH_Aro_), 8.21 (s, 2H, NH_2_) ppm; ^13^C NMR (100 Hz, DMSO-*d*_6_): δ 87.2, 98.0, 113.7, 114.3, 119.0, 125.0, 126.4, 129.4, 132.1, 134.3, 149.3, 155.3, 158.7 ppm.

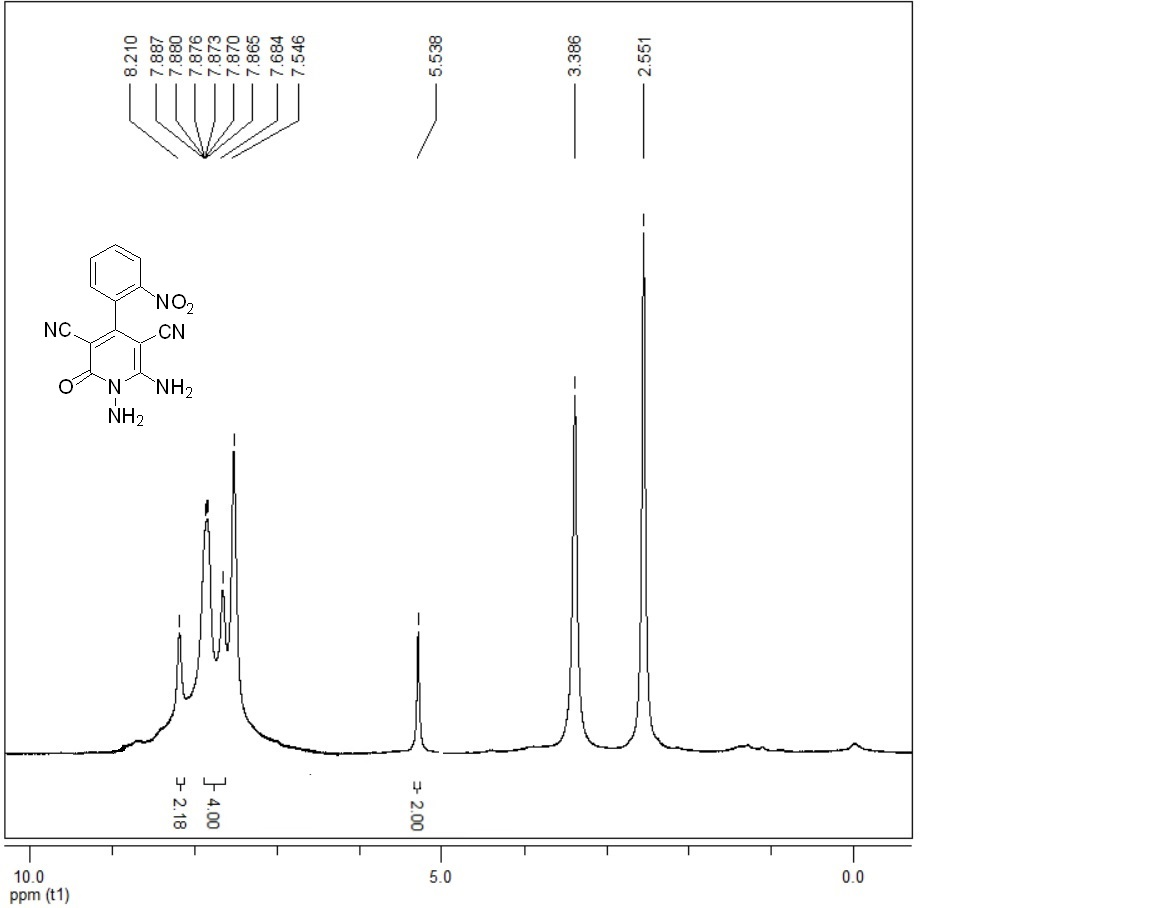


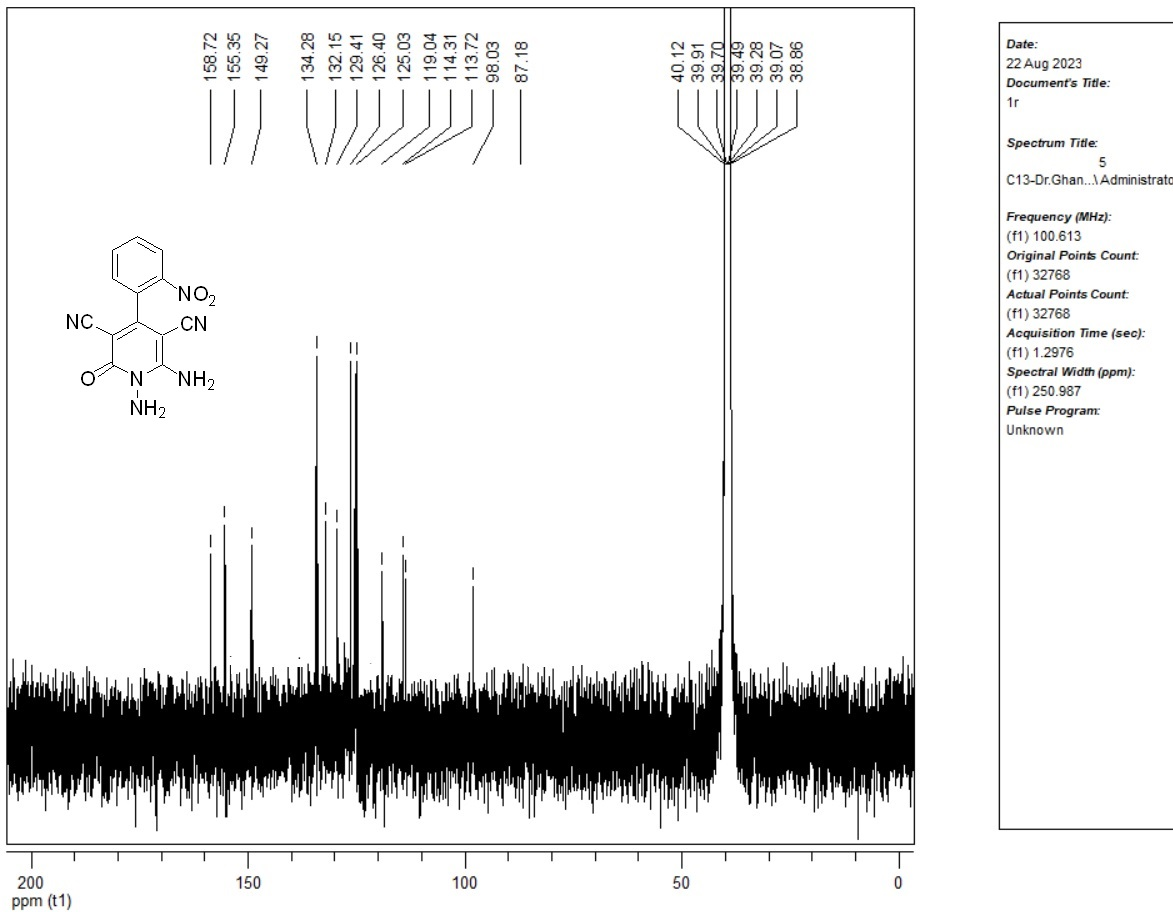


**^1^H NMR and ^13^C NMR of compound (5f)**

*1,6-diamino-2-oxo-4-phenyl-1,2-dihydropyridine-3,5-dicarbonitrile (****5f****)*: m.p = 238-240 °C (lit.^47^ 237-240); Yield: 87 %; ^1^H NMR (400 MHz, DMSO-*d_6_*): δ 5.69 (s, 2H, NH_2_), 7.51-7.63 (m, 5H, CH_Aro_), 8.51 (s, 2H, NH_2_) ppm; ^13^C NMR (100 Hz, DMSO-*d*_6_): δ 74.3, 86.4, 115.4, 116.3, 128.0, 128.6, 130.2, 134.6, 156.6, 159.2, 159.5.

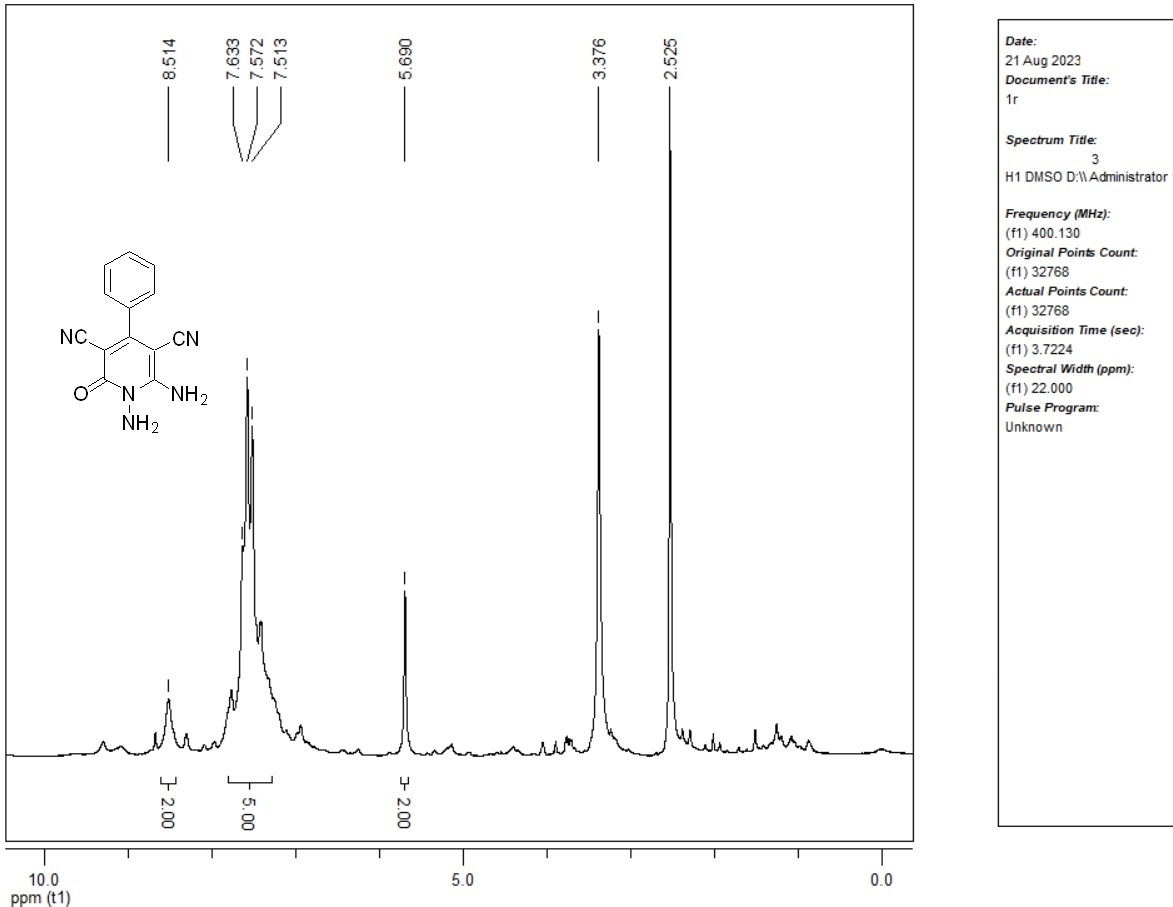


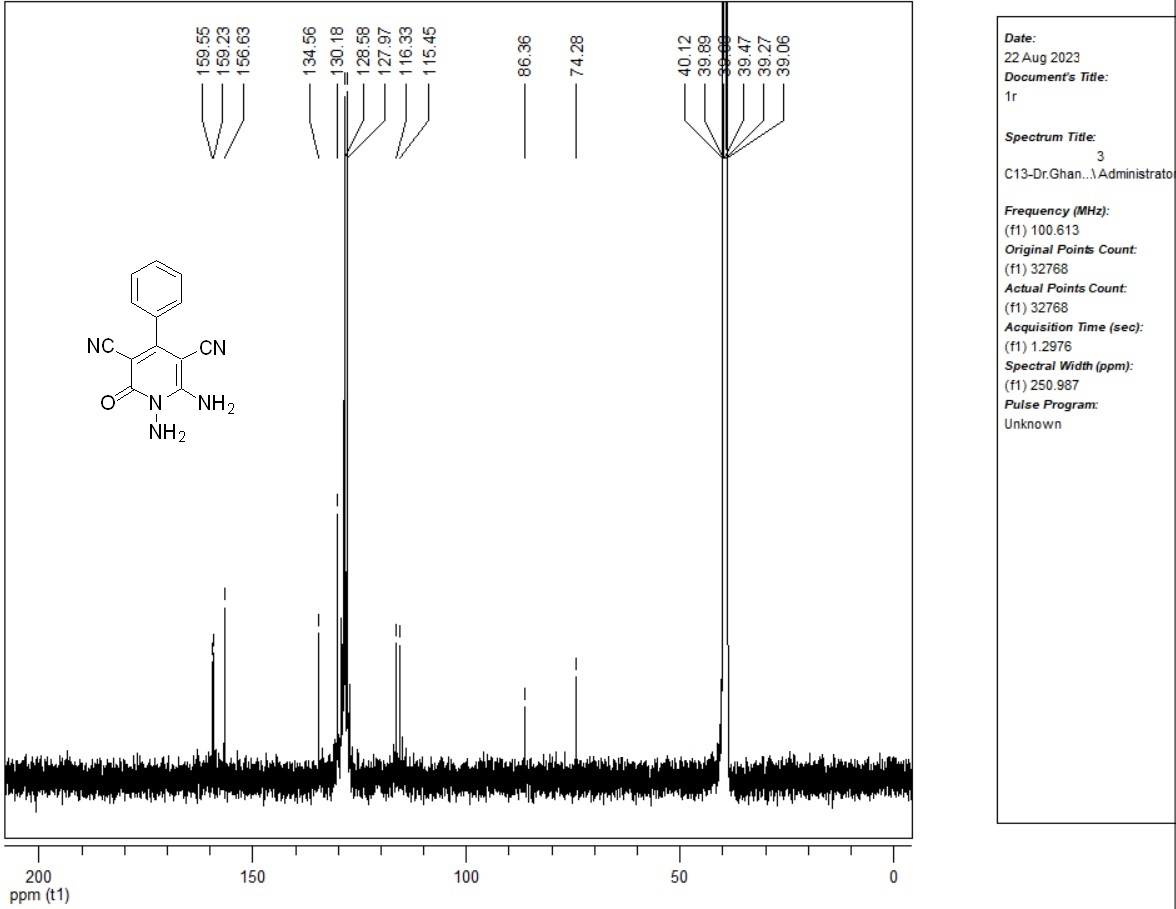


**^1^H NMR and ^13^C NMR of compound (5g)**

*1,6-diamino-4-(4-(dimethylamino)phenyl)-2-oxo-1,2-dihydropyridine-3,5-dicarbonitrile (****5g****)*: m.p = 248-250 °C (lit.^47^ 249-251); Yield: 99 %; ^1^H NMR (400 MHz, DMSO-*d_6_,* ppm) δ: 3.07 (s, 6H, 2CH_3_), 5.67 (s, 2H, NH_2_) ppm, 6.86 (d, 2H, *J*= 8.8 Hz, CH_Aro_), 7.41 (d, 2H, *J*= 8.4 Hz, CH_Aro_), 8.38 (brs, 2H, NH_2_) ppm; ^13^C NMR (100 Hz, DMSO-*d*_6_): δ = 73.6, 85.5, 111.1, 111.7, 116.2, 120.6, 128.9, 129.5, 151.5, 156.7, 159.5, 159.6 ppm.

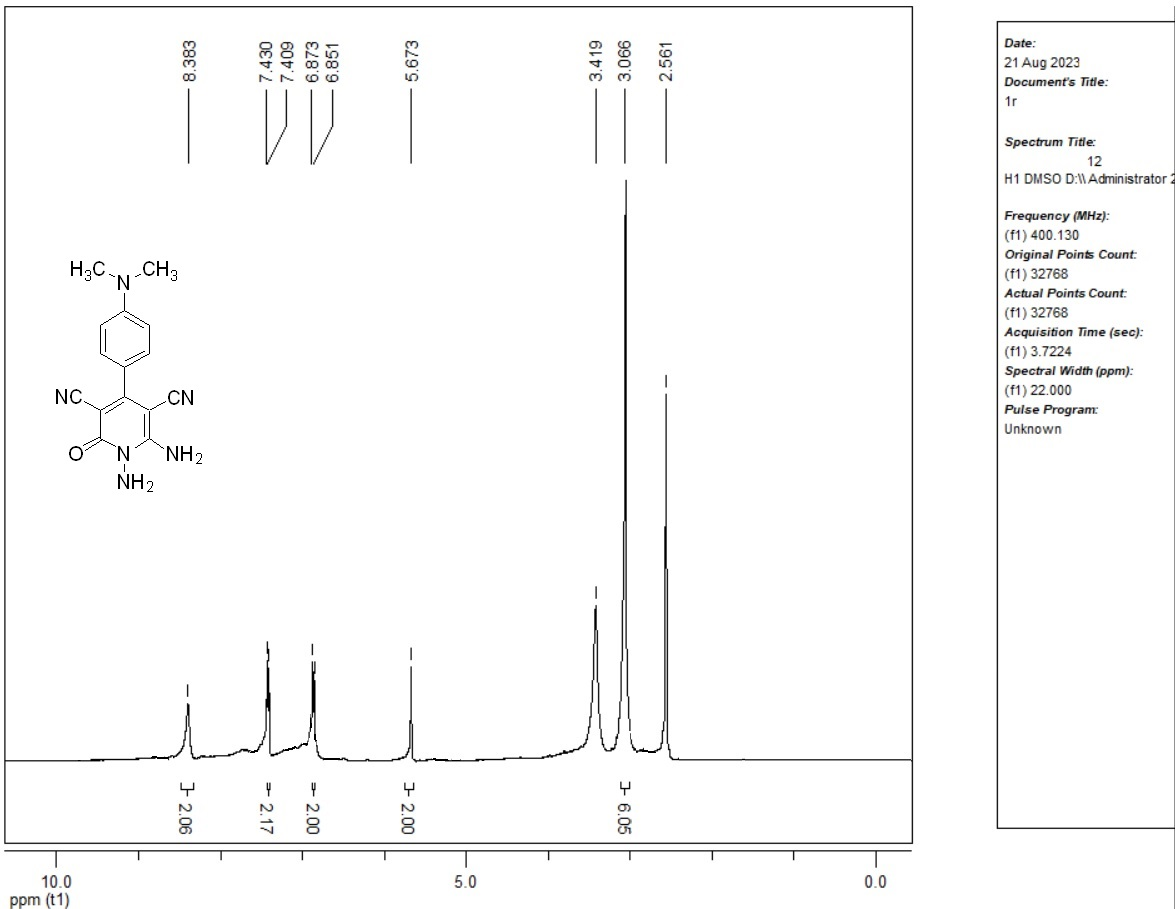


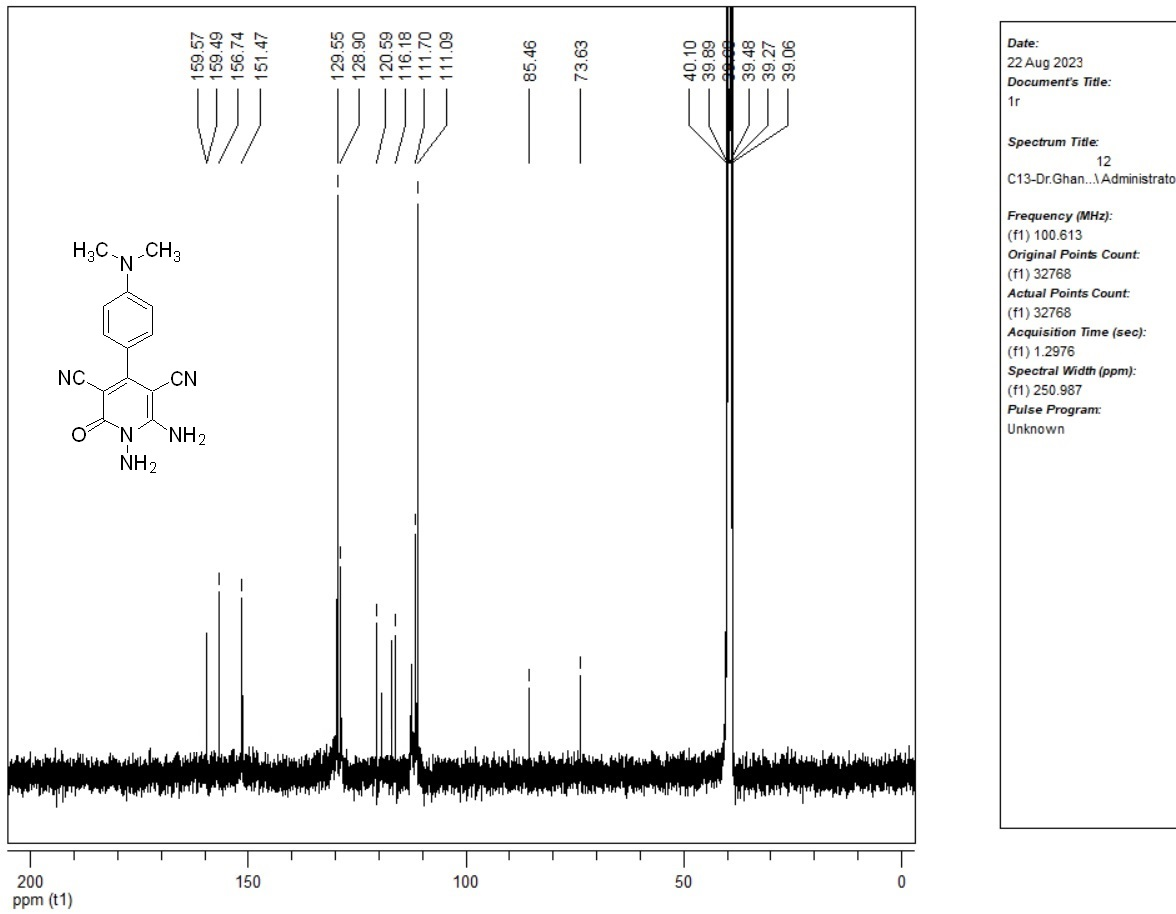


**^1^H NMR and ^13^C NMR of compound (5h)**

*1,6-diamino-4-(4-hydroxyphenyl)-2-oxo-1,2-dihydropyridine-3,5-dicarbonitrile (****5h****)*: m.p: 324-326 °C (lit.^47^ 325-327 °C); Yield: 99 %,; ^1^H NMR (400 MHz, DMSO-*d_6_*) δ: 5.64 (s, 2H, NH_2_), 6.90 (d, 1H, *J*= 8.4 Hz, CH_Aro_), 6.95 (d, 1H, *J*= 8.8 Hz, CH_Aro_), 7.34 (d, 1H, *J*= 8.4 Hz, CH_Aro_), 8.02 (d, *J*= 8.4 Hz, 1H, CH_Aro_), 8.40 (brs, 1H, NH_2_), 10.05 (s, 1H, OH) ppm; ^13^C NMR (100 Hz, DMSO-*d*_6_): δ 74.1, 115.2, 116.4, 122.5, 124.8, 129.9, 134.0, 154.8, 156.6, 159.3, 162.9 ppm.

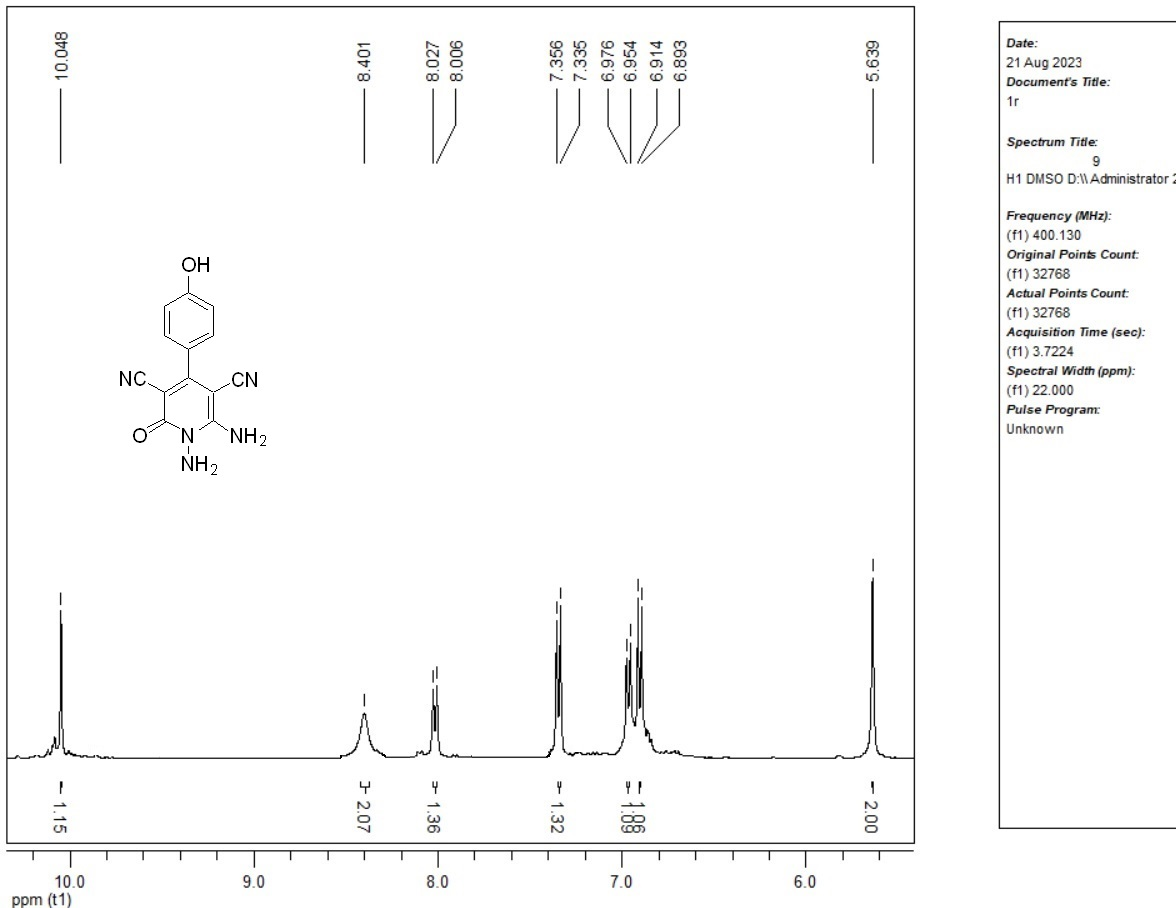


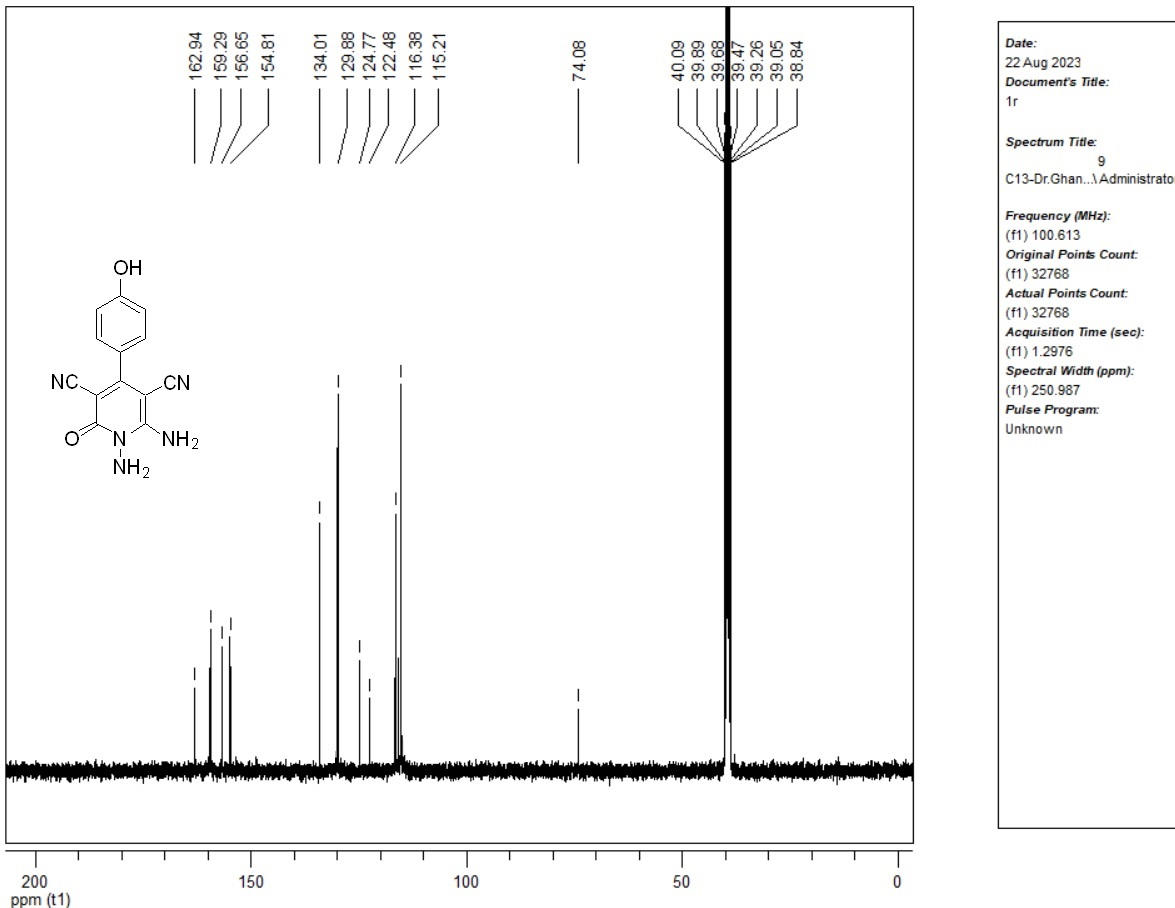


**^1^H NMR and ^13^C NMR of compound (5i)**

*1,6-diamino-4-(3-nitrophenyl)-2-oxo-1,2-dihydropyridine-3,5-dicarbonitrile (****5i****)*: m.p: 250-251 °C (lit.^49^ 249-251 °C); Yield 98 %; ^1^H NMR (400 MHz, DMSO-*d_6_*) δ: 5.68 (s, 2H, N-NH_2_), 6.42 (brs, 2H, H-Ar), 6.66 (brs, 2H, H-Ar), 8.83 (brs, 2H, NH_2_) ppm. ^13^C NMR (100 Hz, DMSO-*d*_6_): δ 76.2, 91.4, 116.7, 125.8, 130.6, 134.4, 135.2, 148.1, 160.3, 160.5, 160.6 ppm.

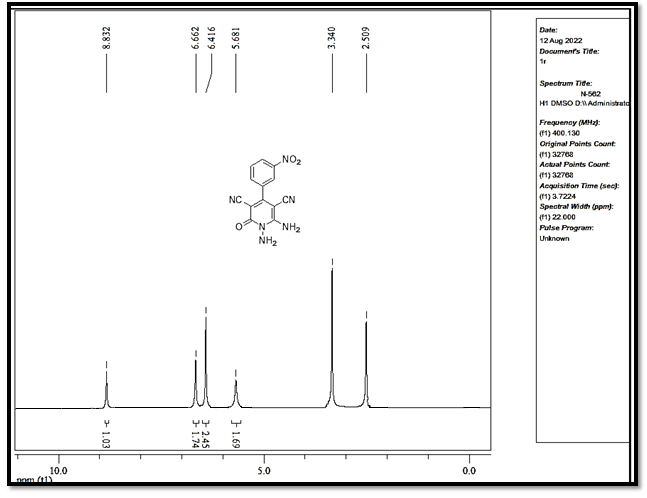


**Fig.1.** ^[1](https://www.google.com/url?sa=t&rct=j&q=&esrc=s&source=web&cd=&cad=rja&uact=8&ved=2ahUKEwjmjuPvw579AhW_8LsIHTvtAd8QFnoECBAQAQ&url=https%3A%2F%2Fwww.researchgate.net%2Ffigure%2FThe-1-H-NMR-spectrum-of-compound-3a-in-CDCl-3-solvent_fig1_320865085&usg=AOvVaw2S68h8geITZVHy7tcJjbIy)^[H NMR spectrum of compound 5j.](https://www.google.com/url?sa=t&rct=j&q=&esrc=s&source=web&cd=&cad=rja&uact=8&ved=2ahUKEwjmjuPvw579AhW_8LsIHTvtAd8QFnoECBAQAQ&url=https%3A%2F%2Fwww.researchgate.net%2Ffigure%2FThe-1-H-NMR-spectrum-of-compound-3a-in-CDCl-3-solvent_fig1_320865085&usg=AOvVaw2S68h8geITZVHy7tcJjbIy)


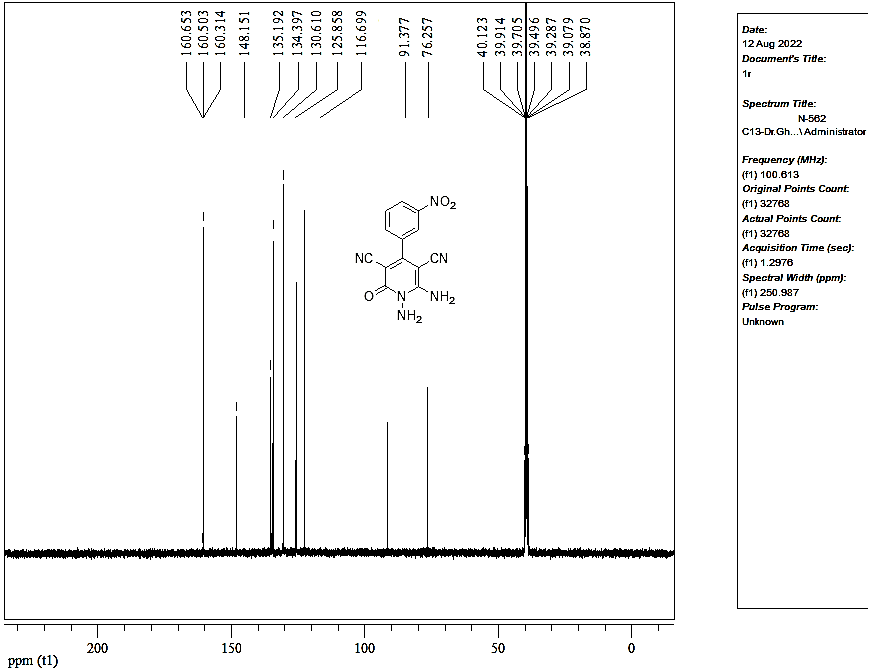


**Fig.1a.**^[13](https://www.google.com/url?sa=t&rct=j&q=&esrc=s&source=web&cd=&cad=rja&uact=8&ved=2ahUKEwjmjuPvw579AhW_8LsIHTvtAd8QFnoECBAQAQ&url=https%3A%2F%2Fwww.researchgate.net%2Ffigure%2FThe-1-H-NMR-spectrum-of-compound-3a-in-CDCl-3-solvent_fig1_320865085&usg=AOvVaw2S68h8geITZVHy7tcJjbIy)^[C NMR spectrum of compound 5j.](https://www.google.com/url?sa=t&rct=j&q=&esrc=s&source=web&cd=&cad=rja&uact=8&ved=2ahUKEwjmjuPvw579AhW_8LsIHTvtAd8QFnoECBAQAQ&url=https%3A%2F%2Fwww.researchgate.net%2Ffigure%2FThe-1-H-NMR-spectrum-of-compound-3a-in-CDCl-3-solvent_fig1_320865085&usg=AOvVaw2S68h8geITZVHy7tcJjbIy)

**^1^H NMR and ^13^C NMR of compound (5j)**

*1,6-diamino-4-(2,4-dimethoxyphenyl)-2-oxo-1,2-dihydropyridine-3,5-dicarbonitrile (****5j****)*: m.p: 252-253 °C (lit.^42^ 251-253); Yield: 99%, ^1^H NMR (400 MHz, DMSO-*d_6_*): δ 3.62 (s, 3H, OCH_3_), 3.85 (s, 3H, OCH_3_), 5.51 (S, 2H, NH_2_), 6.66-7.24 (m, 3H, CH_Aro_), 8.85 (s, 2H, NH_2_) ppm; ^13^C NMR (100 Hz, DMSO-*d*_6_): δ 55.1, 55.5, 66.1, 105.0, 118.9, 119.4, 130.7, 132.0, 159.3, 161.4, 162.2, 164.9, 174.6 ppm.

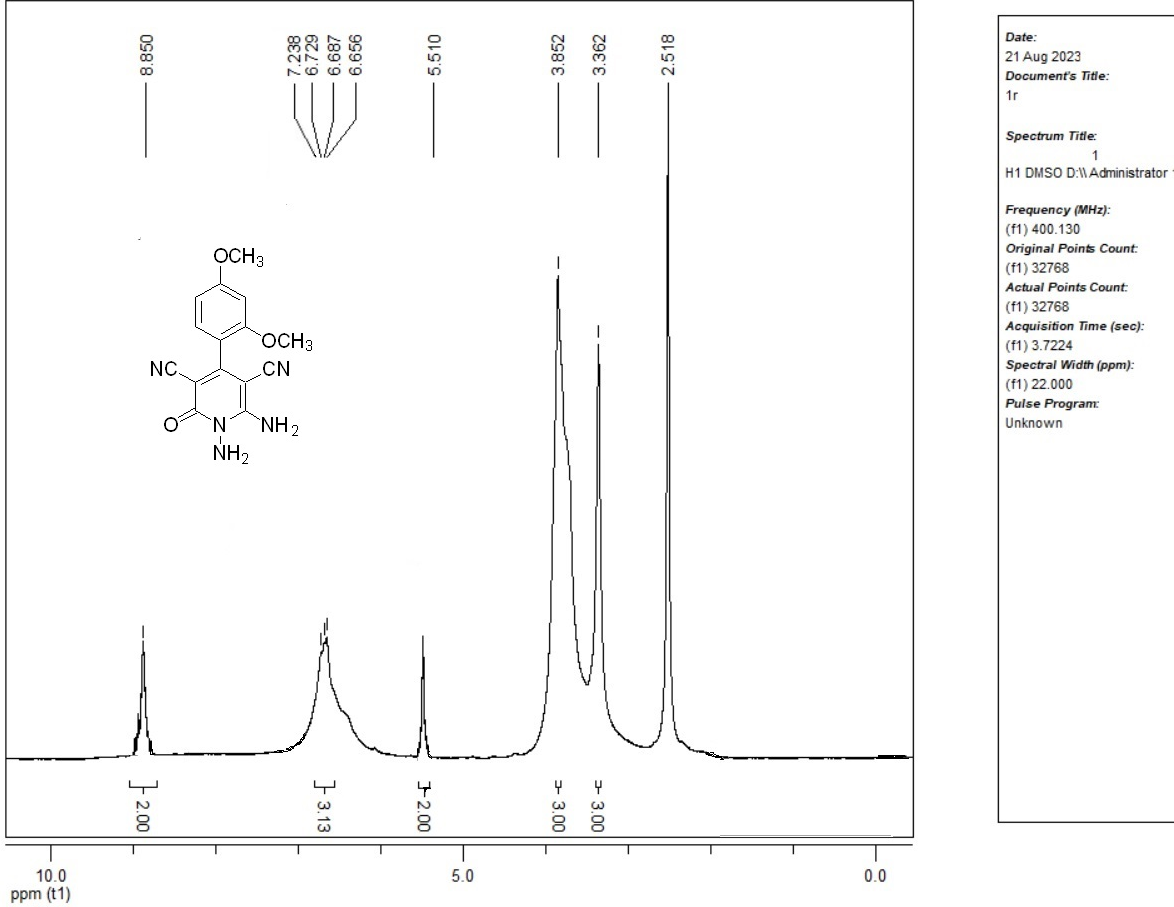


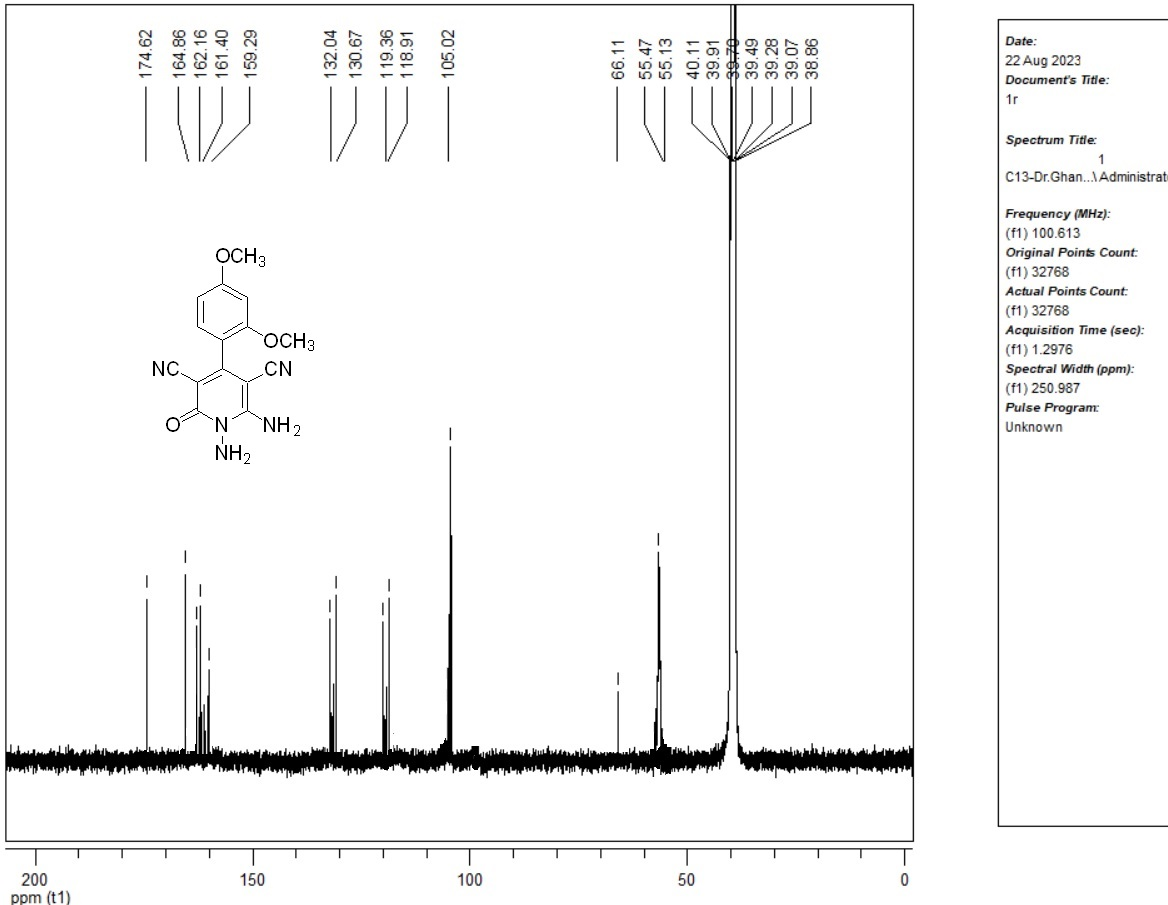


**^1^H NMR and ^13^C NMR of compound (5k)**

*1,6-diamino-2-oxo-4-(p-tolyl)-1,2-dihydropyridine-3,5-dicarbonitrile (****5k****)*: m.p: 238-240 °C (lit.^47^ 240-241°C); Yield: 97 %; ^1^H NMR (400 MHz, DMSO-*d_6_*): δ 5.66 (s, 2H, NH_2_), 7.35-7.40 (m, 4H, CH_Aro_), 8.45 (brs, 2H, NH_2_) ppm. ^13^C NMR (100 Hz, DMSO-*d*_6_): δ 20.9, 74.2, 86.3, 115.5, 116.4, 127.9, 129.1, 131.6, 140.0, 156.6, 159.3, 159.6 ppm.

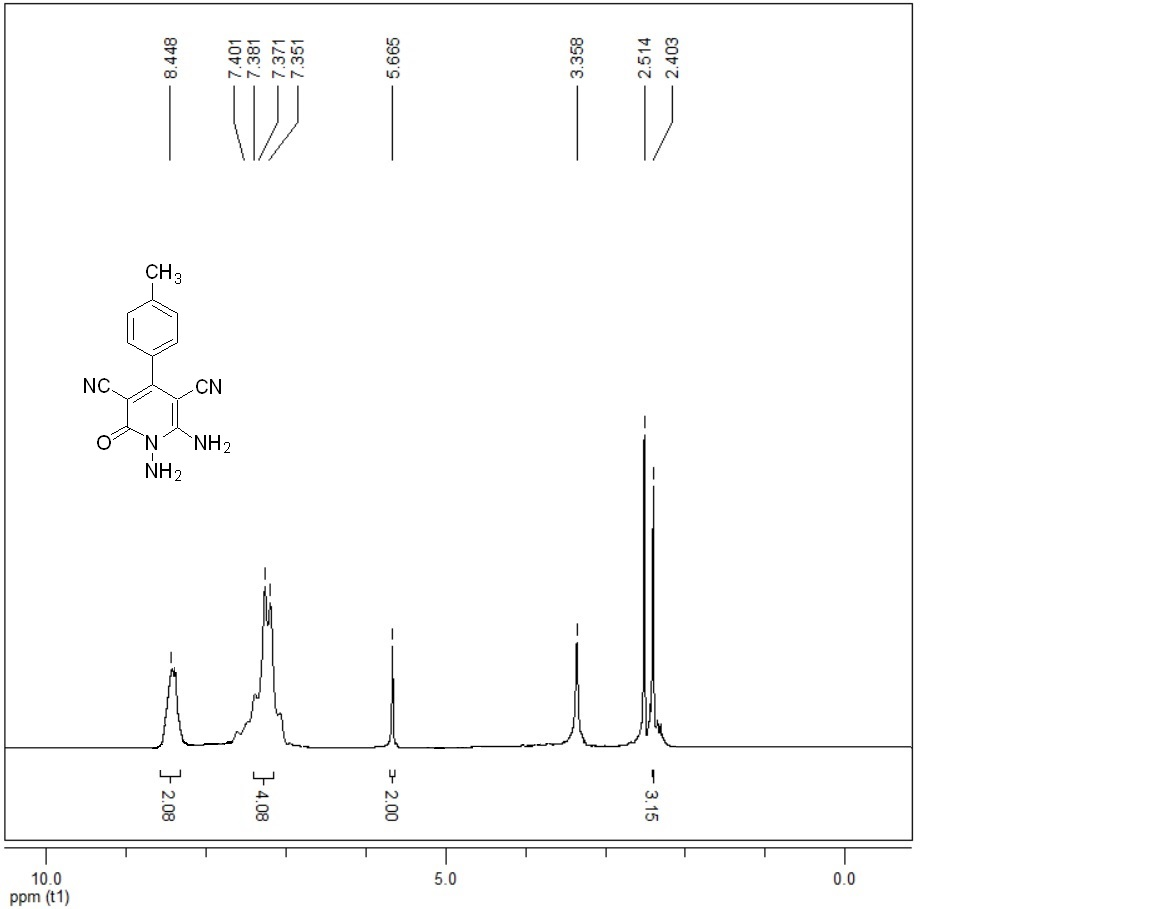


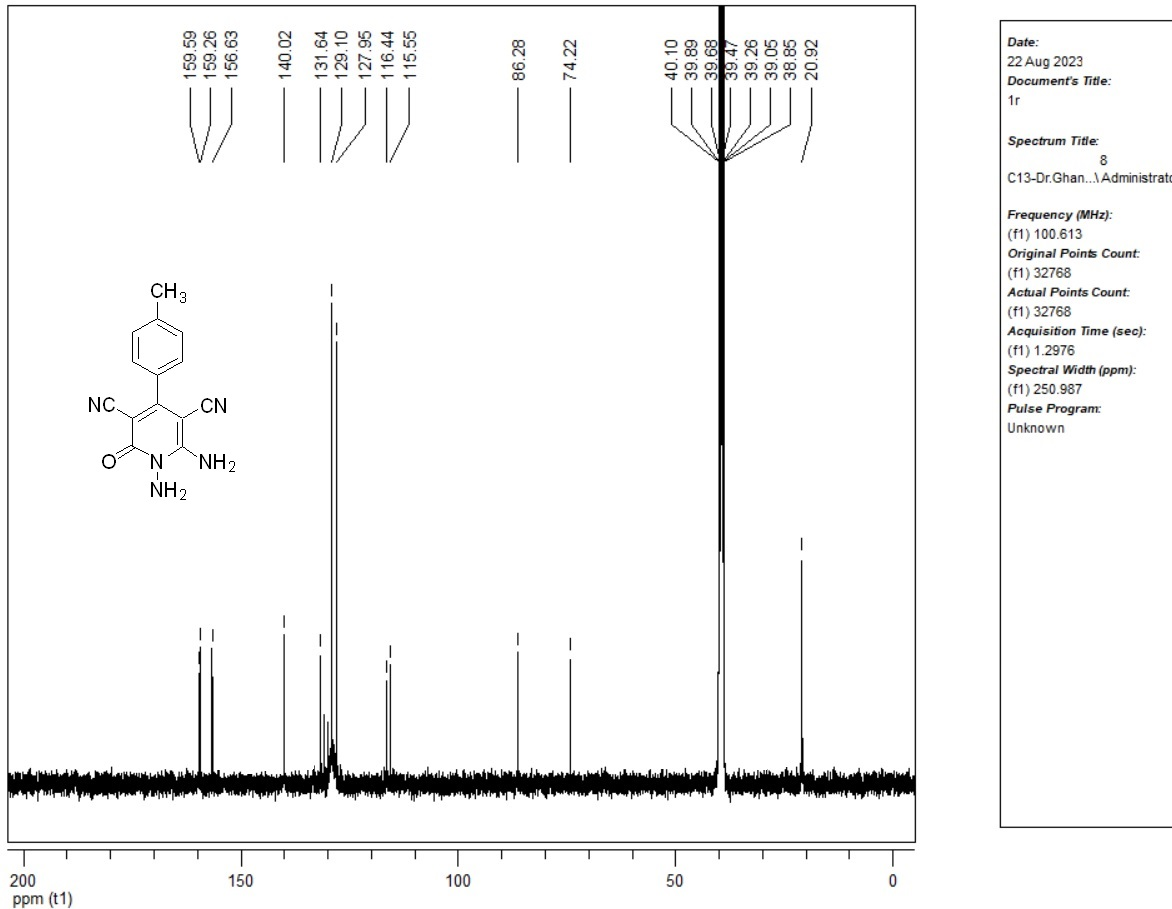


**^1^H NMR and ^13^C NMR of compound (5l)**

*1,6-diamino-4-(furan-2-yl)-2-oxo-1,2-dihydropyridine-3,5-dicarbonitrile (****5l****)*: m.p: 323-326 °C (lit.^47^ 325-327 °C); Yield: 98 %; ^1^H NMR (400 MHz, DMSO-*d_6_,*) δ: 5.79 (s, 2H, NH_2_), 7.49-7.62 (m, 3H, CH_Aro_), 8.17 (s, 2H, NH_2_) ppm; ^13^C NMR (100 Hz, DMSO-*d*_6_): δ = 80.0, 114.4, 137.4, 139.2, 143.5, 146.5, 148.2, 150.2, 155.9, 164.1, 171.6 ppm.

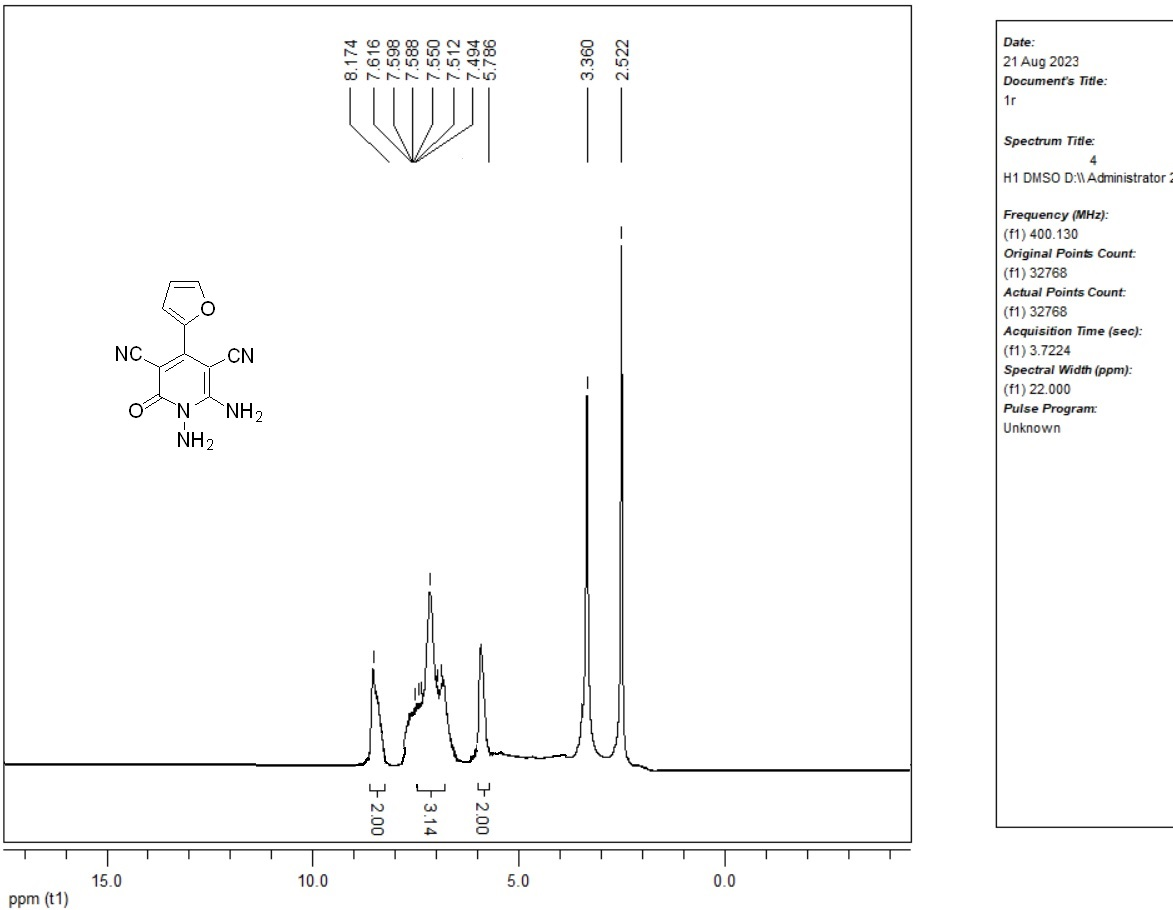


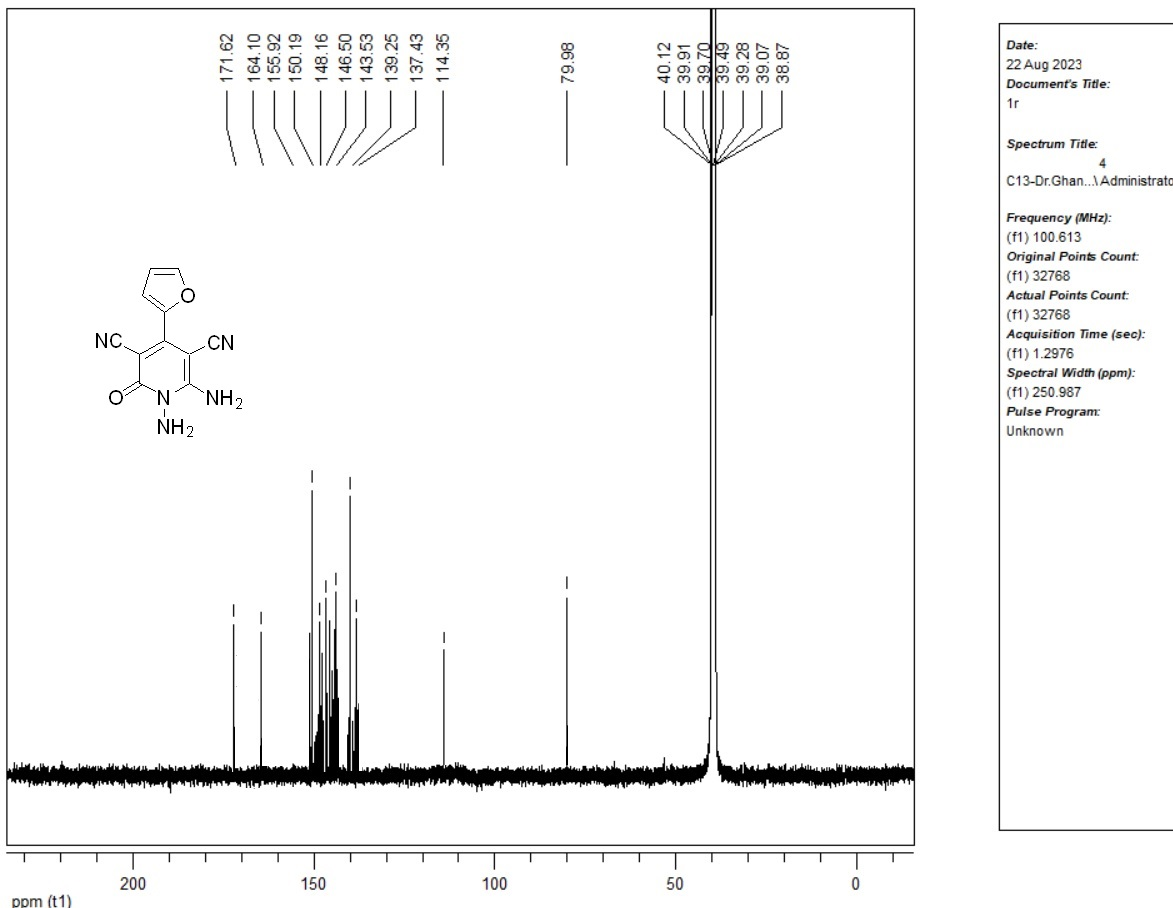


**^1^H NMR and ^13^C NMR of compound (5m)**

*1',6'-diamino-2'-oxo-1',2'-dihydro-[2,4'-bipyridine]-3',5'-dicarbonitrile (****5m****)*: m.p: 296-297 °C; Yield: 90 %; ^1^H NMR (400 MHz, DMSO-*d_6_*): δ 5.79 (s, 2H, NH_2_), 7.64-7.88 (m, 4H, CH_Aro_), 8.81 (s, 2H, NH_2_) ppm; ^13^C NMR (100 Hz, DMSO-*d*_6_): δ 85.2, 113.9, 121.8, 123.7, 129.6, 134.7, 140.9, 156.5, 161.6, 183.7, 187.7, 188.2 ppm.

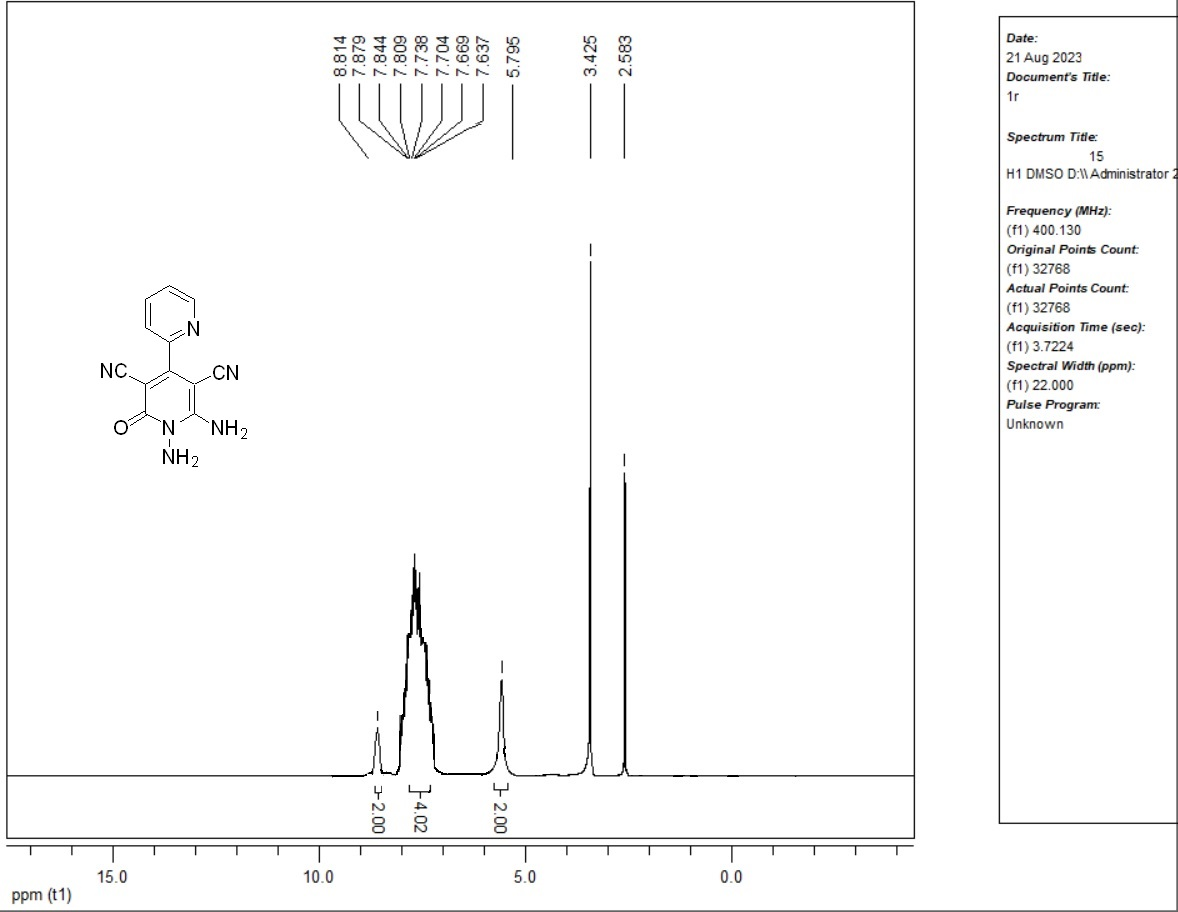


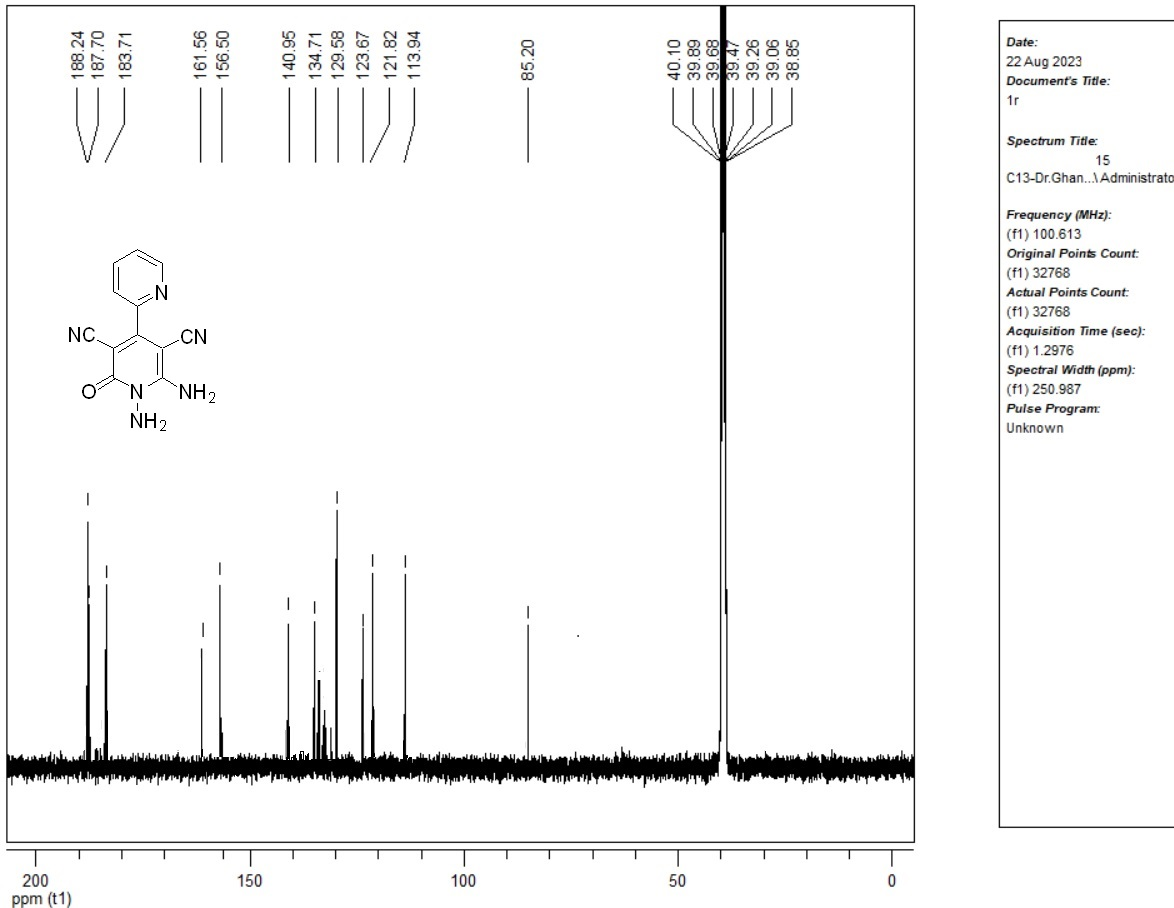

Supplement: Supplementary file 1 — Supplementary Information. [file 41598_2023_43045_MOESM1_ESM.docx]
